# Multiomics Landscape Uncovers the Molecular Mechanism of the Malignant Evolution of Lung Adenocarcinoma Cells to Chronic Low Dose Cadmium Exposure

**DOI:** 10.3389/fonc.2021.654687

**Published:** 2021-11-11

**Authors:** Shun-Dong Dai, Shuang Wang, Ya-Nan Qin, Jin-Chao Zhu

**Affiliations:** ^1^ Department of Pathology, Shanghai Ninth People’s Hospital, Shanghai JiaoTong University School of Medicine, Shanghai, China; ^2^ Department of Pathology, Shenyang Red Cross Hospital, Shenyang, China

**Keywords:** cadmium, chronic exposure, lung adenocarcinoma, whole-exome sequencing (WES), RNA sequencing (RNAseq)

## Abstract

Cadmium (Cd) from cigarette smoke and polluted air can lead to lung adenocarcinoma after long-term inhalation. However, most studies are based on short-term exposure to this toxic metal at high concentrations. Here, we investigate the effects of long-term exposure of A549 cells (lung adenocarcinoma) to cadmium at low concentrations using morphological and multiomics analyses. First, we treated A549 cells continuously with CdCl_2_ at 1μM for 8 months and found that CdCl_2_ promoted cellular migration and invasion. After that, we applied transmission electron and fluorescence microscopies and did not observe significant morphological changes in Golgi apparatus, endoplasmic reticulum, lysosomes, or mitochondria on Cd treated cells; microfilaments, in contrast, accumulated in lamellipodium and adhesion plaques, which suggested that Cd enhanced cellular activity. Second, by using whole-exome sequencing (WES) we detected 4222 unique SNPs in Cd-treated cells, which included 382 unique non-synonymous mutation sites. The corresponding mutated genes, after GO and KEGG enrichments, were involved mainly in cell adhesion, movement, and metabolic pathways. Third, by RNA-seq analysis, we showed that 1250 genes (784 up and 466 down), 1623 mRNAs (1023 up and 591 down), and 679 lncRNAs (375 up and 304 down) were expressed differently. Furthermore, GO enrichment of these RNA-seq results suggested that most differentially expressed genes were related to cell adhesion and organization of the extracellular matrix in biological process terms; KEGG enrichment revealed that the differentially expressed genes took part in 26 pathways, among which the metabolic pathway was the most significant. These findings could be important for unveiling mechanisms of Cd-related cancers and for developing cancer therapies in the future.

## Introduction

Cadmium (Cd) is one of the known toxic and carcinogenic transition metals that is distributed widely in the environment. It was classified as a class I human carcinogen by the International Agency for Research on Cancer (IARC) in 1993 (1). The lung is a primary target organ of exposure to cadmium because this metal is mainly absorbed through inhalation (2, 3). Cadmium has a very long biological half-life, which result in accumulative toxic and carcinogenic effects (4). Therefore, the harm to the human body also shows long-term and multifaceted characteristics (3). However, most previous studies described the potential of cadmium to cause carcinogenic effects using short-term exposure to this toxic metal at high concentrations (5, 6). To mimic conditions more similar to occupational and cadmium exposure that is relevant biologically (7), we exposed human lung adenocarcinoma cell line A549 at a concentration of 1μM for 8 months in our study.

Pulmonary cancer is one of the leading malignant tumors in the world. Despite, the existence of various chemo/physical/immunological therapies, pulmonary cancer in many countries still has a low 5-year survival rate (<15%) (8). Indeed, epidemiological investigations (using specific pollution) and experimental studies (using laboratory animals) have both demonstrated that Cd exposure increased the risk of pulmonary adenocarcinomas (3, 9). However, the specific mechanism of how cadmium exposure promoted the development of lung cancer has not been documented. Carcinogenesis is a multi-stage process that involves a multitude of alterations to the cell. Several studies have focused on how cadmium exposure induced malignant degeneration of bronchial epithelial cells or alveolar epithelial cells, but cadmium promotion of cancer development is involved in all stages of cancer development. There is still a lack of research on how cadmium exposure promotes the malignant progression of lung cancer. In our study, human lung A549 cells were chosen because they display many differentiated features of lung alveolar cells and have been used by many researchers in the cadmium toxicity studies (10–12).

With the advent of next generation sequencing technologies, recent carcinogenic investigations have focused on studying global exome and transcriptome changes to understand the molecular basis of cancer (13, 14). Omics data, such as exome, transcriptomics, and epigenomics, provide us an integrated global functional view on cellular responses after exposure to environmental substances. These responses can be linked to the generation and progression of disease. Genome/exome-wide data sets, a complication of large, curated, mechanistic databases, and bioinformatic predictions can deepen our understanding of the sequence of events; these understandings can help us to propose a hypothesis, which can be validated using experimental approaches (15). In this study, we aim to investigate somatic genetic alterations of adenocarcinoma (A549 cells) after long-term, low dose cadmium exposure by using whole-exome sequencing (WES) and RNA-sequencing analyses.

## Results

### Effects of Chronic Low Dose Cadmium Exposure on Cytoskeleton, Mitochondria, Endoplasmic Reticulum, Golgi Apparatus, and Lysosomes of A549 Cells

To determine the influence of cadmium (Cd) on A549 cells, we performed the following experiments: 1) verification of the identity of the A549 cell line; and 2) imaging the cellular ultrastructure and organelle morphology. DNA fingerprinting using short tandem repeat (STR) profiling is an easy and reliable tool that can be used to verify cell lines. It does not change significantly with cell passage number (16). For verification, we confirmed the identity of A459 cells and excluded cross-contamination possibilities in long-term cell culture, by profiling DNA fingerprinting with the STR (**Supplementary Figure 1**). The results showed that Cd-treated (A549+Cd) cells and Cd-untreated (A549+H0) cells had essentially identical profiles, which indicated no cross-contamination in these two cell lines.

**Figure 1:**
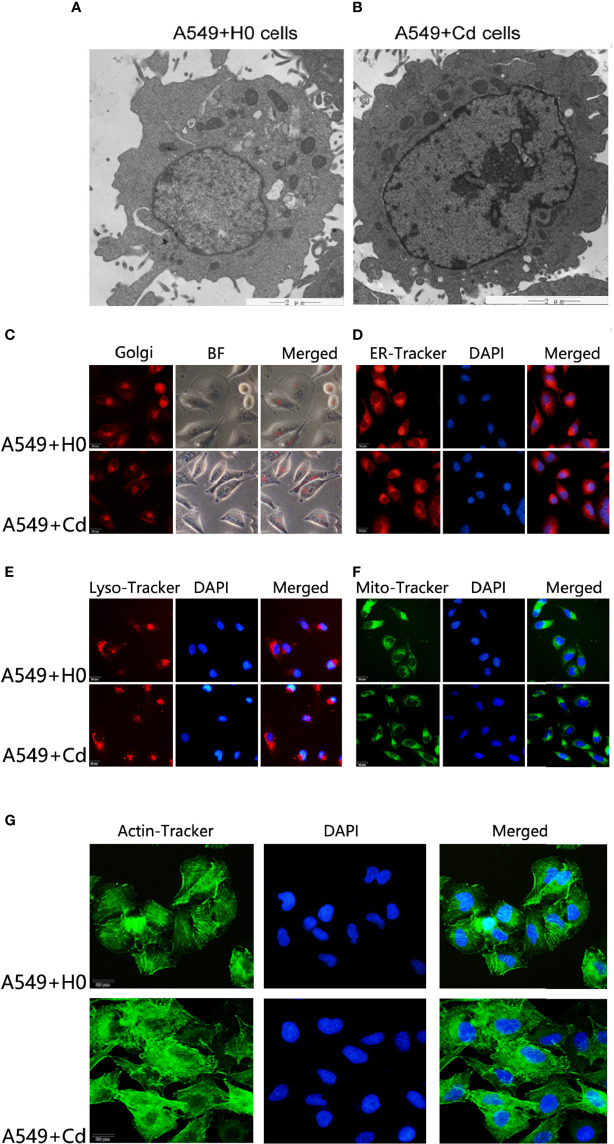
Morphology of cellular organelles and cytoskeleton after Cd exposure. Intracellular structures of A549 cells were imagined by a transmission electron microscope (TEM) after chronic low-dose cadmium (Cd) exposure. **(A, B)** Ultrastructure comparisons of A549 cells: A549+H0 as blank compared with A549+Cd as Cd treatments. **(C–F)** No significant morphological changes were found in Golgi apparatus, endoplasmic reticulum (ER), lysosomes (LysoE), or mitochondria (Mito) after Cd treatments. **(G)** Cytoskeleton disorder was observed in Cd treatment group (A549+Cd).

A great deal of evidence suggests that the biphasic nature of cadmium is characterized by low-dose stimulation and high-dose inhibition (17, 18).To evaluate this hypothesis in our experimental system, we treated A549 cells with a low concentration (1µM) of cadmium chloride (CdCl_2_) for 8 months. For ultrastructure imaging, we used a transmission electron microscope (TEM) and found that long-term exposure to CdCl_2_ did not change the ultrastructure of A549 cells noticeably (**Figures 1A, B**), which suggested Cd did not exhibit a biphasic nature on A549 cells in this setting.

For imaging of organelle morphology, we probed the mitochondria, the endoplasmic reticulum (ER), the Golgi, and lysosomes with fluorescent dyes. No significant morphological change was found in Golgi apparatus, endoplasmic reticulum, lysosomes, or mitochondria after the long-term exposure to Cd (**Figures 1C**–**F**). However, when we probed the actin cytoskeleton, we found that, actin was apparently more disordered after Cd treatment (**Figure 1G**). The cytoskeleton is a complex of detergent-insoluble components of the cytoplasm that play critical roles in cell motility, shape generation, and mechanical properties of a cell. These results implied that long-term exposure to low doses of CdCl_2_ might affect organization of A549 cells in the actin cytoskeleton.

### Promoting Adenocarcinoma A549 Migration and Invasion

**Figure 2:**
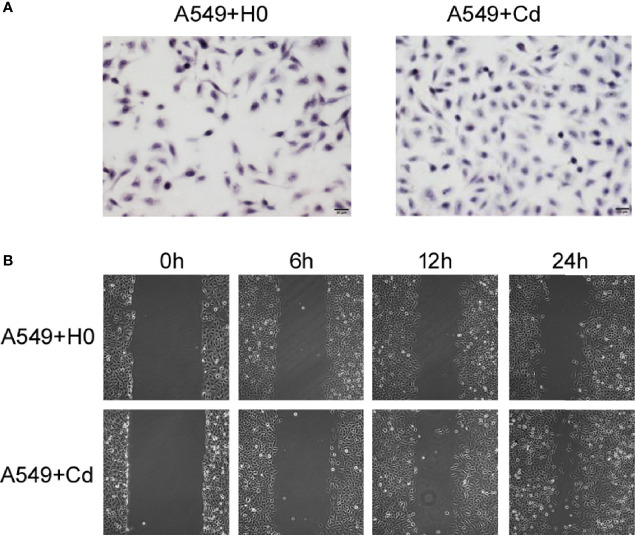
Invasion and migration of A549 cells. **(A)** Cell invasion was analyzed by the Matrigel Transwell assay. Cd exposure promoted cell invasive behaviors as indicated by more purple cells in the A549+Cd group. **(B)** Cell migration assays showed narrow gaps in A549+Cd group, which indicated that Cd can promoted the immigration capacity of A549 cells.

To examine the effects of chronic low dose CdCl_2_ on migration and invasion of A549 cells, we performed the Transwell invasion assay and the Scratch test migration assay. Our invasion assay showed that Cd exposure increased the cell numbers of invasion; the cell numbers of A549+Cd group and A549+H0 group were 144.3 ± 7.41 and 98.5 ± 12.86((t=4.965, p=0.0157), respectively (**Figure 2A**). The migration assay showed that A549+Cd cells exhibited increased migration ability compared with A549+H0 cells, as demonstrated by the narrower scratching gap between cells (**Figure 2B**). These results indicated that chronic cadmium exposure, even at a low dose, may affect cell invasion and migration.

### Genomic Variations in A549 Cells Due to Chronic Low Dose Cadmium Exposure

**Figure 3:**
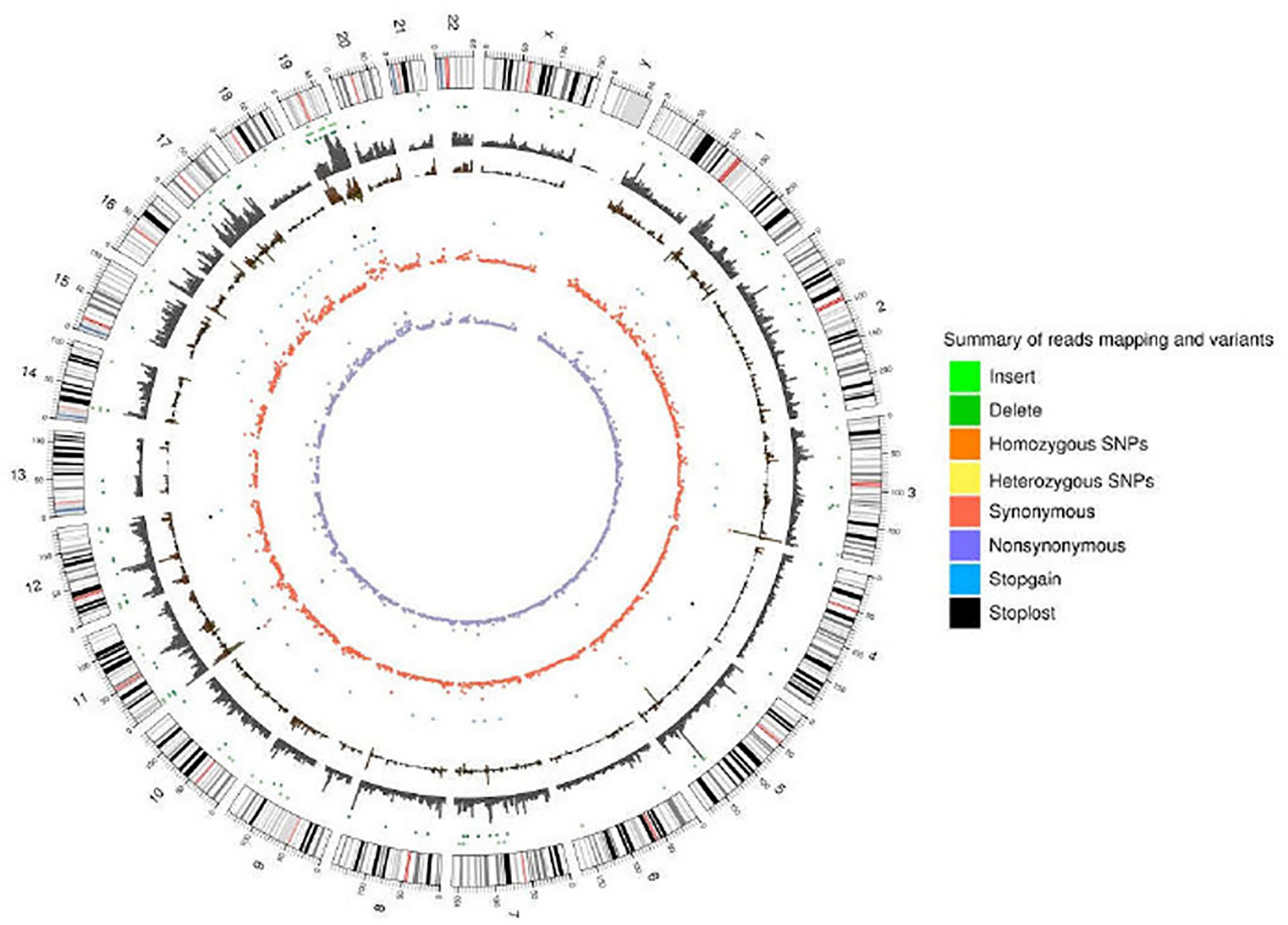
Reads coverage of each chromosome and the statistical results of variation loci.

As a significant development of next-generation sequencing, WES is a powerful tool for evaluating genomic variation. To identify genomic variation between the A549+Cd cells and A549+H0 cells, we performed high- through put, WES, which generated a total of ~33.0 Gb of data (>20× depth, 87× depth max) (**Table 1**). Based on the sequencing data, we characterized a total of 58,289 SNPs in A549+Cd cells and 50,887 SNPs in A549+H0 cells (**Figure 3** and **Supplemental Data 1**).

**Table 1:** Exon capture statistic of A549+H0 cells and A549+Cd cells.

Exon Capture Statistics	A549+H0 cells DNA	A549+Cd cells DNA
**Target size(bp)**	62,085,295	62,085,295
**Effective reads**	69,157,446	57,625,498
**Aligned reads**	67,779,635	56,391,115
**%Aligned reads**	98.01	97.86
**Reads in target region**	34,499,846	29,664,782
**%Reads in target region**	50.90	52.61
**Mean depth of target region**	83.35	71.67
**Coverage of target region**	97.9247275865	97.5160655998
**Reads in flanking region**	24,521,219	20,989,258
**%Reads in flanking region**	36.18	37.22
**Mean depth of flanking region**	51.18	43.81
**Coverage of flanking region**	95.67	93.39
**%Targeted region covered at depths of at least 4X**	97.81	97.38
**%Targeted region covered at depths of at least 10 X**	97.38	96.75
**%Targeted region covered at depths of at least 20X**	96.25	94.94
**%Flanking region covered at depths of at least 4X**	94.92	92.37
**%Flanking region covered at depths of at least 10X**	92.00	88.11
**%Flanking region covered at depths of at least 20X**	84.22	76.81
**Non-duplicated reads**	55,532,142	47,132,701
**%Non-duplicated reads**	80.30	81.79
**Uniq mapped reads**	48,971,224	41,493,173
**%Uniq mapped reads**	70.81	72.00

**Figure 4:**
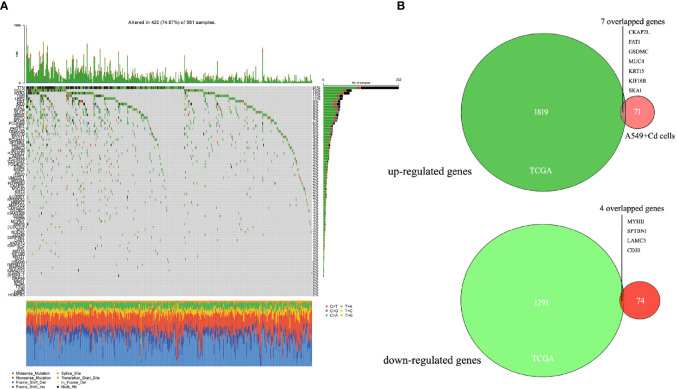
Seventy-eight mutated genes associated with cell migration and invasion were identified in the TCGA lung adenocarcinoma database. **(A)** Waterfall of 78 mutant genes in TCGA lung adenocarcinoma. The most frequently mutated genes were TTN (41%), PCLO (16%), RYR3(14%) and LRP2 (11%). **(B)** Venn diagrams of overlapping up-regulated genes and down-regulated genes in TCGA database and A549+Cd cells.

For further biological analyses, we selected 6876 non-synonymous mutation SNP sites, 125 non-frameshift mutations, 64 frameshift mutations, 52 terminator acquisition loci, and six terminator loss loci from both cells. Subsequently, 382 unique non-synonymous mutation SNPs were detected in the A549+Cd cells (**Supplemental Data 2**) compared with A549+H0 cells. Among these 382 SNPs, 78 affected genes were associated with cell migration and invasion ability. However, none of them harbored a specific pathogenic mutation based on rigorous evaluation (Pathogenicity Calculator, http://calculator.clinicalgenome.org/site/cg-calculator) (**Supplemental Data 3**). To explore the affected 78 genes in human lung adenocarcinoma, we downloaded human lung adenocarcinoma SNP mutation data and transcriptome data (normal: 54, tumor: 497) (TCGA-LUAD) from a TCGA database. We found that the mutation rates of TTN, PCLO, RYR3, and LRP2 in human lung adenocarcinoma were 41%, 16%, 14% and 11%, respectively, but the mutation rates of other mutant genes were <10% (**Figure 4A** and **Table 2**). From the DESeq differential analysis, 1826 up-regulated genes and 1295 down-regulated genes were obtained from transcriptome date of TCGA lung adenocarcinoma. By taking the intersection as shown in **Figure 4B**, the expressions of CKAP2L, FAT1, GSDMC, MUC4, KRT15, KIF18B and SKA1 of 78 affected genes were up-regulated in the TCGA database, MYH11, SPTBN1, LAMC3 and CD33 were down-regulated, and other genes were not expressed differentially in lung adenocarcinoma of TCGA.

**Table 2:** Overlapping mutation genes in A549+Cd cells and in the TCGA lung adenocarcinoma database.

Up-regulated	p-value	q-value	SNP number	Down-regulated	p-value		q-value		SNP number
CKAP2L	4.26E-12	9.89E-11	10	MYH11	4.52E-21		3.02E-19	42	
FAT1	3.37E-05	0.000244209	58	SPTBN1	9.62E-18		4.64E-16	27	
GSDMC	2.76E-09	4.26E-08	10	LAMC3	6.34E-10		1.08E-08	33	
MUC4	9.01E-11	1.76E-09	16	CD33	0.001136		0.00566129	16	
KRT15	0.010675	0.038861457	6						
KIF18B	1.59E-16	6.50E-15	4						
SKA1	2.63E-10	4.75E-09	3						

**Figure 5:**
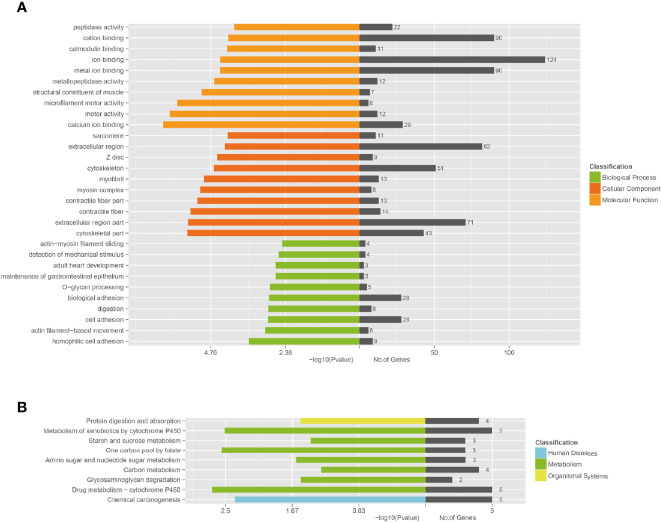
Functional enrichment of unique mutated genes. **(A)** The Gene Ontology (GO) enrichment of unique mutated genes in A549+Cd cells. GO analysis comprises three branches: cellular components, molecular functions, and biological processes. **(B)** The Kyoto Encyclopedia of Genes and Genomes (KEGG) pathway analysis of unique mutated genes in A549+Cd cells. KEGG analysis includes three branches: human diseases, metabolisms, and organismal systems.

Functional annotation analysis of genes in the associated regions was performed using different databases, which included NCBI non-redundant (NR), The Gene Ontology (GO), Clusters of Orthologous Groups (COG), and Kyoto Encyclopedia of Genes and Genomes (KEGG) Pathway analysis. According to GO enrichment analysis, the unique mutant genes were enriched in 30 GO terms, which included 10 biological process terms (BP), 10 molecular function terms (MF), and 10 cellular component terms (CC). In BP terms, most of the mutated gene enrichments, involved adhesion and cell movement, such as biological adhesion, cell adhesion, homophilic cell adhesion, and actin filament-based movement (**Table 3** and **Figure 5**). In MF terms, mutated genes were largely related to metal ion binding, cation binding, and calcium ion binding. In CC terms, major mutations were involved in extracellular regions and the cytoskeleton.

**Table 3:** Gene Ontology(GO) enrichment analysis of mutated genes in A549+Cd cells.

GOMFID	Term	Gene Names
Molecular Function		
GO:0005516	calmodulin binding	TTN;RYR3;OBSCN;MYO7A;MYO5A;MYH4;MYO10;MYH7;AEBP1;MYH11;SPTBN1
GO:0008237	metallopeptidase activity	ADAMTS7;MMP8;ADAMTS17;ERAP1;DNPEP;MMP27;ADAMTSL3;ADAMTS1;CPB2;ASTL;AEBP1;CPA4
GO:0000146	microfilament motor activity	MYO3B;MYO7A;MYO5A;MYH4;MYO10;MYH7
GO:0005509	calcium ion binding	PKD1L2;DST;ANXA13;PCDHA8;PCDHA4;MMP27;FAT1;RYR3;UMODL1;GPR98;EMR3;MMP8;CD248;CUBN;HRNR;LRP2;PCDH8;PCDHGC5;TTN;NID1;PCLO;PITPNM3;MYO5A;PCDHB12;PCDHGA7;PCDHB8;SCUBE1;EML1;ITSN2
GO:0003774	motor activity	KIF2B;DNAH6;MYO7B;MYO3B;MYO7A;MYO5A;MYH4;MYO10;MYH7;DNAH10;MYH11;KIF18B
Cellular Component		
GO:0005576	extracellular region	PKD1;ADAMTS7;IMPG1;DST;ANXA13;ACAN;KRT15;ALDH3A1;PSMB1;TPSAB1;MMP27;FAT1;ZNF559-ZNF177;PLEC;EMR3;UPK3A;CUBN;PPL;SERPINI1;WDR60;NEB;BTBD17;ACR;SLC1A5;ADAMTS17;C17orf99;SLC12A3;ERAP1;PODN;A2M;CLEC18C;GPR98;RSPO2;TIAM2;ZNF177;ZNF446;CPA4;ADAMTS1;CD248;LAMC3;VWA8;KLK1;MMP8;INHA;ITGAV;FABP1;CCDC147;DNPEP;ITSN2;ADAMTSL3;HRNR;AP4M1;LRP2;APOA1BP;AEBP1;PNP;GSTP1;UMODL1;SPTBN1;TTN;NID1;CHIA;VASN;RAB3GAP1;KLK13;SORL1;TPSB2;PGC;EPN3;OTOP1;TNN;ENO3;MUC5B;MUC6;CPB2;MUC4;JUP;SSPO;CCDC105;SCUBE1;MUC2;SLC9A3
GO:0005856	cytoskeleton	KIF2B;MNS1;GSDMC;GTF2E1;CCDC105;DST;TTLL6;KRT15;ROCK2;KIAA0753;MAP2K5;MYO10;PLEC;PDE4DIP;RADIL;PPL;KRTAP9-4;NEB;SFI1;MYO3B;DNAH6;TRIP6;KIAA0368;HOMER3;CAST;KRTAP4-1;KRT3;KIF18B;MYO7A;MAP7D3;MCPH1;MYH4;PCLO;JUP;MYH7;MYH11;CKAP2L;SPTBN1;TTN;MYO7B;KRTAP10-2;TMEM214;SKA1;KRT27;ITPR1;MYO5A;RPS6KA2;DNAH10;MAP1B;EML1;HRNR
GO:0016459	myosin complex	MYO7B;MYO3B;MYO7A;MYO5A;MYH4;MYO10;MYH7;MYH11
GO:0044430	cytoskeletal part	KIF2B;MNS1;GSDMC;CCDC105;DST;TTLL6;KRT15;ROCK2;KIAA0753;MAP2K5;MYO10;DNAH10;PDE4DIP;RADIL;KIAA0368;SFI1;MYO3B;DNAH6;KRTAP9-4;HOMER3;KRTAP4-1;KRT3;KIF18B;MYO7A;MAP7D3;MCPH1;MYH4;JUP;MYH7;MYH11;CKAP2L;SPTBN1;TTN;MYO7B;KRTAP10-2;TMEM214;SKA1;KRT27;ITPR1;MYO5A;RPS6KA2;MAP1B;EML1
Biological Process		
GO:0007156	homophilic cell adhesion	PKD1;PCDHA8;PCDHA4;FAT1;PCDHGA7;PCDHB8;PCDHGC5;PCDH8;PCDHB12
GO:0030048	actin filament-based movement	TTN;NEB;MYO7A;MYO5A;MYH4;MYH7
GO:0007155	cell adhesion	DST;PCDHA8;PCDHA4;MAP2K5;MYO10;FAT1;ACAN;RADIL;SERPINI1;PKD1;TRIP6;GPR98;LAMC3;JUP;ASTL;AEBP1;PCDH8;ROCK2;PCDHGC5;NID1;CD33;ITGAV;TNN;PCDHB12;PCDHGA7;MUC4;PCDHB8;SSPO
GO:0022610	biological adhesion	DST;PCDHA8;PCDHA4;MAP2K5;MYO10;FAT1;ACAN;RADIL;SERPINI1;PKD1;TRIP6;GPR98;LAMC3;JUP;ASTL;AEBP1;PCDH8;ROCK2;PCDHGC5;NID1;CD33;ITGAV;TNN;PCDHB12;PCDHGA7;MUC4;PCDHB8;SSPO
GO:0033275	actin-myosin filament sliding	TTN;MYH4;NEB;MYH7

The KEGG enrichment revealed that the unique mutant genes in A549+Cd cells were enriched in nine pathways, in which the metabolic pathway had the majority of enriched genes (**Figure 5A**). The enrichment analysis showed that unique mutant genes in A549+Cd cells were associated with protein digestion and absorption (four mutated genes), metabolism of xenobiotics by cytochrome P450 (five mutated genes), starch and sucrose metabolism(three mutated genes), one carbon pool by folate (three mutated genes), amino sugar and nucleotide sugar metabolism genes (three mutated genes), carbon metabolism (four mutated genes), glycosaminoglycan degradation (two mutated genes), drug metabolism-cytochrome P450 (five mutated genes), and chemical carcinogenesis (five mutated genes) (**Figure 5B**).

### Profiles of Transcriptomes Due to Chronic Low Dose Cadmium Exposure of A549 Cells to Cadmium

To characterize transcriptomic differences after long-term exposure to Cd, we conducted next-generation RNA sequencing. Two groups of cells were sequenced: 1) A549+Cd cells with 51,201 transcripts (32,223 mRNAs and 18,979 lncRNAs); and 2) A549+H0 cells with 51,136 transcripts (31,925 mRNAs and 19,211 lncRNA) (**Supplementary Data 4**).

By comparing the RNA sequencing data of A549+Cd and A549+H0 cells, we obtained the differential profile transcriptomes that were caused by chronic, low dose, cadmium exposure. We identified 1250 differentially expressed genes, among which 784 genes were significantly up-regulated and 466 genes were down-regulated in A549+Cd cells, compared with A549+H0 cells. Among them, 132 differentially expressed genes were related to migration; 85 up-regulated genes (**Table 4**) and 47 down-regulated genes (**Table 5**). We also identified differentially expressed mRNAs, which included 1023 increased and point 591 decreased mRNAs. Transcriptional data also indicated that there were 679 differentially expressed lncRNAs(375 increased and 304 decreased).

**Table 4:** Differentially up-regulated genes associated with cell migration and invasion in A549+Cd cells.

Gene	log2FoldChange	q-value	Gene	log2FoldChange	q-value
JAG2	1.20	6.47E-15	PCDHA3	1.17	6.47E-15
ANXA13	1.36	0.000233	MMP17	2.31	5.10E-15
FBLN5	2.40	5.10E-15	DGKA	1.71	1.11E-09
TNC	1.33	5.10E-15	CALB2	1.14	5.72E-08
TLL1	1.43	1.72E-11	TLL2	1.31	0.000299
NCALD	4.03	6.47E-15	THBS1	1.03	6.47E-15
SCUBE2	1.87	1.64E-05	PCDH20	2.62	2.76E-06
EMR1	1.02	5.10E-15	RASGRP4	1.14	4.90E-05
PCDHGA2	1.05	9.09E-13	RCN3	1.05	3.59E-08
PCDHAC2	1.10	6.47E-15	CDH22	3.23	6.83E-06
C1S	1.14	8.99E-15	C1R	1.63	8.99E-15
PADI3	2.45	5.10E-15	PCDHGB3	1.11	5.10E-15
PCDHA4	1.06	6.47E-15	SPARC	3.02	5.10E-15
NPNT	3.02	9.74E-08	PCDHA10	1.00	5.10E-15
DSC3	2.55	6.47E-15	SPOCK2	1.32	9.09E-05
PROCA1	1.02	4.55E-10	CDH4	1.25	6.47E-15
PCDH7	1.01	0	NPHS1	1.27	6.14E-07
REPS2	1.12	8.99E-15	ELTD1	4.46	9.09E-06
PRRG4	1.20	5.10E-15	CDK5R1	1.29	7.81E-11
PCDHB5	2.41	5.10E-15	DLL1	1.02	6.75E-07
CLDN2	1.50	5.10E-15	VAV3	1.12	6.47E-15
RND1	1.73	1.59E-11	CDH16	1.87	0
THEMIS2	1.35	1.03E-07	ANGPT1	1.74	1.70E-06
FES	1.46	0.000669	DSCAML1	2.65	5.10E-15
NRARP	1.03	1.95E-10	CLDN3	4.06	5.10E-15
CD4	3.57	1.94E-05	EMB	2.31	6.47E-15
HMCN1	1.64	0	NOTCH3	2.65	0
LRRN2	2.55	0	AMIGO1	1.17	1.18E-09
PDGFRA	1.31	5.66E-05	TNFAIP6	1.79	1.15E-05
ITGA4	2.58	6.47E-15	ICAM1	1.26	0
CADM2	4.72	5.21E-07	ESAM	2.40	5.59E-13
COL1A1	1.06	4.32E-14	EPHA7	4.91	6.47E-15
NEO1	1.07	6.47E-15	AMBP	1.58	5.10E-15
SAA1	3.53	5.10E-15	ITGB6	2.19	0
PTPRD	1.24	0	C1QTNF1	3.15	0
AZU1	1.38	7.55E-05	SERPINF2	2.76	6.47E-15
HOXA7	1.40	0.000593	CCL5	3.06	5.10E-15
ITGAX	1.91	1.93E-10	TRO	2.78	5.10E-15
BCAM	1.41	6.47E-15	EDA	1.94	7.64E-13
KANK1	1.18	8.22E-15	MCAM	1.35	0
NTN1	1.21	1.39E-08	LAMC2	2.18	6.47E-15
STAT5A	1.22	5.47E-07	GREM1	3.21	1.71E-07
NLGN4Y	2.23	5.10E-15			

**Table 5:** Differentially down-regulated genes associated with cell migration and invasion ability in A549+Cd cells.

Gene	log2FoldChange	q-value	Gene	log2FoldChange	q-value
SYT1	-1.00	6.47E-15	MMP19	-1.29	9.35E-15
EFCAB12	-1.54	0.000499	SVEP1	-1.71	1.25E-99
ANXA8L1	-1.12	3.95E-05	FBN2	-1.18	1.78E-37
EFEMP1	-4.82	1.19E-304	EDIL3	-2.24	0
PC	-1.18	1.78E-90	MMP16	-1.31	5.65E-28
FAT3	-1.31	3.66E-113	CD248	-2.58	0.000567
CDH2	-4.77	0	CCBE1	-3.48	5.59E-33
EFEMP2	-1.09	3.20E-22	FSTL4	-3.77	4.10E-195
EFHD1	-1.18	1.62E-05	COL13A1	-1.29	3.68E-19
CYP1B1	-3.19	0	COL12A1	-2.11	1.27E-234
CYR61	-1.49	1.19E-174	ICAM5	-1.18	2.86E-07
HAS2	-1.88	1.80E-06	MSLN	-2.06	0.000122
FOXC2	-3.18	0.000930	VEGFA	-1.17	7.98E-303
HAPLN2	-1.04	0.000876	TENM3	-1.39	4.13E-209
NLGN1	-1.35	1.06E-25	ITGB3	-1.73	0
CD226	-2.21	1.51E-09	BMP2	-3.35	0.000270
CXCR7	-2.70	6.46E-18	DLC1	-2.97	1.92E-22
PTK2B	-1.04	1.05E-45	IL32	-1.25	9.70E-05
SELPLG	-1.53	2.83E-06	FPR2	-4.83	1.08E-13
WISP2	-2.57	0	EDIL3	-2.24	0
THY1	-3.58	4.05E-05	CNTN1	-2.25	0
ITGBL1	-3.73	1.93E-172	COL4A6	-2.23	2.09E-141
WNT5A	-1.99	5.11E-64	CLDN16	-1.54	1.99E-05
CLDN4	-1.04	1.90E-13			

Functional enrichment analysis based on GO annotations showed that the differentially expressed genes of A549+Cd cells were enriched in 30 GO terms, which included 10 biological process (BP) terms, 10 molecular function (MF) terms, and 10 cellular component (CC) terms (**Supplementary Data 5** and **Figure 5**). Most differentially expressed genes that were enriched in BP terms may be related to cell adhesion and extracellular matrix organization, such as biological adhesion, cell adhesion, which was similar to the GO annotations based on WES. Most differentially expressed genes enriched in MF terms were related to receptor binding and protein heterodimerization activity. In addition, most differentially expressed genes enriched in CC terms were related to extracellular regions and integral/intrinsic components of plasma membrane. These results from the transcriptomes were similar to the results of the entire exome, which indicated that Cd long-term exposure may affect development of carcinoma.

**Figure 6:**
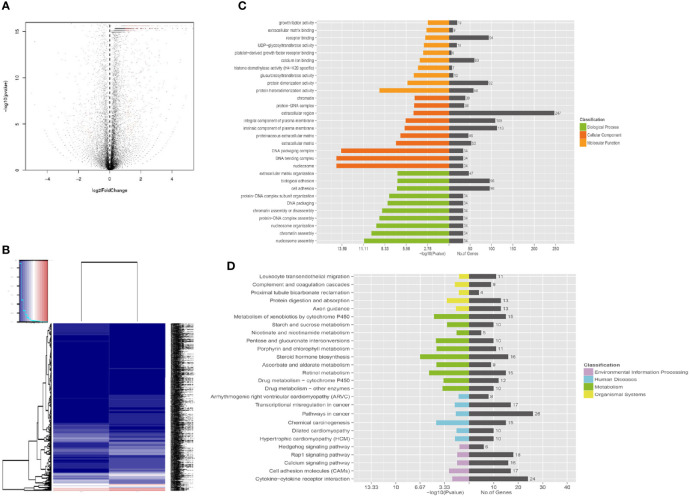
Functional enrichment of different expression genes. **(A)** Volcano map of gene expression analysis between A549+Cd cells and A549+H0 cells. **(B)** Heat map of gene expression analysis. **(C)** The Gene Ontology (GO) enrichment of differentially expressed genes. GO analysis contains three branches: cellular components, molecular functions, and biological processes. **(D)** The Kyoto Encyclopedia of Genes and Genomes (KEGG) pathway analysis of differentially expressed genes. KEGG analysis includes four branches: environmental information processing, human diseases, metabolisms, and organismal systems.

Apart from GO annotations, we also performed KEGG enrichment analysis and found that the DEGs were involved in 26 pathways (**Supplementary Data 6**); the metabolic pathway was the most significant (**Figure 6**). Other significant pathways were chemical carcinogenesis, transcriptional mis-regulation in cancer, cytokine-cytokine receptor interaction, calcium signal pathway, Rap1 signal pathway and Hedgehog pathway.

## Discussion

Cadmium (Cd) is a heavy element that is known to accumulate in the body and to have strong biological effects. The main entry for cadmium into the body include the respiratory tract, digestive tract, and skin contact. As a non-essential element with 20-30 years half-life *in vivo*, the Cd can remain in the human kidney and liver for almost a lifetime. Therefore, the harm to the human body also shows long-term and multifaceted characteristics. Because the relationship between cadmium and “pain disease” was put forward in the 1960s, there have been many studies on the relationship between environmental pollution and human health due to cadmium. Due to daily-life and industrial activities, 4000 to 13000 tons of Cd has been discharged into the environment every year (19). These toxic pollutants can then accumulate in the human body through direct inhalation of air, drinking water, or by eating crops that grew in polluted soil (19). In addition, occupational exposure and smoking are also important avenues (20). Unlike the kidney and liver, the lung is a primary target organ, directly exposed to Cd pollutants, especially for those people with occupational exposure or for those who smoke tobacco. The affected lung can suffer acute inflammation, chronic edema, bronchitis, and even cancers (21). For the underlying molecular mechanisms of these diseases, Cd may be a strong inducer for deletion of multiple loci in the genes (or gene mutations). Because it cannot be bound to DNA stably, Cd might lead to gene mutations by indirect genotoxic mechanisms, such as oxidative stress, inhibition of DNA repair, stimulation of cell proliferation, blockage of apoptosis, and epigenetic mechanisms (4, 22).

Although Cd can induce gene mutation indirectly and promote the development of cancer, a global picture of the progression of adenocarcinoma after long-term exposure to low-dose Cd is still vague. Previous studies have reported that cadmium affected the biological behavior of cells through Wnt (23, 24), PI3K/AKT (25, 26) or JNK (5, 27) signaling pathways, but there is still a lack of studies on the effect of long-term, low dose cadmium exposure on whole exome and transcriptomes of lung cancer cells in the lung. Cadmium itself has the ability to induce gene mutation indirectly (28) and, therefore, it inhibits the DNA repair system, which results in gene damage and accumulation of mutation. To provide a clear picture, we sequenced whole exome and transcriptomes of A549 cells after a low dose, long-term treatment with Cd and then analyzed the function of the mutated genes by GO and KEGG enrichments. Our exome results showed that 78 mutated genes were related to biological processes of cell movement and migration, such as calmodulin binding, microfilament movement function, calcium binding function, cytoskeleton composition, actin-related biological processes, and cell adhesion. Our transcriptomic results showed that Cd affected the expression of genes related to calcium binding, cell adhesion, DNA, and protein synthesis. These influences are signs of transcriptomic disorders in tumor progression. The transcriptomic disorders may lead further to cytoskeleton disorders in A549 cells and then affect biological functions, such as adhesion and migration. Because Cd was shown previously to regulate Wnt, PI3K/AKT and JNK signaling pathways, we speculated that these pathways together with the disorders found in our studies rendered progression of adenocarcinoma more malignant. Other tumor-related pathways (e.g., Hedgehog pathway, Rap1 signal pathway, and calcium signal pathway, and chemical carcinogenesis) might also crosstalk with those disorders.

Reactive oxygen species (ROS) can be induced by Cd-related cellular disorders, such as decreasing the activity of antioxidant enzymes, imbalance of intracellular calcium homeostasis, interference with cellular calcium metabolism, and damage of the DNA repair mechanism (29, 30). Over the last decade, many investigators have highlighted the involvement of ROS-stimulated signaling in metal-induced carcinogenesis. Unlike general concepts on the relationship between metal and ROS, a direct role of ROS is not considered likely in cadmium-induced carcinogenesis because cadmium does not participate in Fenton-type chemical reactions. Although cadmium is not a direct mutagenic inducer, proposed mechanisms of cadmium-induced carcinogenesis include formation of ROS, alteration of antioxidant enzymes, inhibition of DNA repair enzymes, and an imbalance between pro- and anti-apoptotic proteins. However, based on our sequencing data, we did not detect obvious alteration of ROS-stimulated signaling, apoptotic signaling, or DNA repair signaling. Thus, we believe that with respect to chronic low dose cadmium exposure, ROS may not play an important role in the malignant progression of cancer. Although ROS might not be involved, we found that metabolic pathways played a critical role in Cd-induced lung cancer progression.

Several studies have identified extensive differences in metabolic profiles between cancerous and normal lung tissue (31, 32), which are consistent with our observations. Alterations in various metabolic pathways conferred selective advantages to cancer cells, such as producing energy and substrates for biosynthesis, increasing redox imbalance, and promoting progression of cancer (e.g., growth, proliferation, and migration) (33). Metabolic toxicity of Cd was rarely discussed in previous studies, because only a few studies indicated that Cd disrupted multiple metabolic pathways in adipocytes and progenitor cells (34).

Several limitations of this study are acknowledged here. First, Cd-induced progression of adenocarcinoma was investigated in A549 cells, which might be different from that measured in lung cancer tissues. Although most of the target genes screened in our cell experiments were validated in a lung cancer cohort, applying the results from surrogate tissue to humans should be done with caution and more advanced models or human tissues are needed to confirm these alterations in the responding genome and transcriptomes. Second, the Cd dose used in this study might not represent the ordinary exposure of human lung tissue. It is difficult to determine the appropriate dose because the reported cadmium levels in lung tissue were largely inconsistent (35–37). To solve this issue, we carried out preliminary experiments and determined suitable conditions that affected the biological behavior of cells significantly without decreasing cell viability. These conditions well mimicked the conditions of chronic Cd exposure and accumulation in lung tissue. Besides, freedom from *in vivo* environmental pressure, especially the tumor surveillance mechanism that removes, morbid cells, may also bias the results. Finally, we only carried out experiments using A549 cells, which acted as surrogate tissue for KRAS mutant/EGFR wild type lung carcinoma. Because the A549 cell line is a KRAS-mutated lung cancer cell line already, the variants may have generated spontaneously from the *in vitro* tissue culture. We did not analyze the changes between the long term cultured A549 and the baseline cell or the low dose exposed A549 cells, which may undermine the impact of long-term culture to the cell line. To better understand the evolution of genomic, transcriptomic and behavioral events at different time points, the short-term, -high dose cadmium exposure model should be included in future studies. Given that lung cancer is highly heterogenous, various lung cancer cell lines or tissues should be utilized to explore the association between Cd-induced, genomic-/transcriptomic- alterations and lung cancer more thoroughly.

In conclusion, Cd as a heavy toxic mental from polluted air or cigarette smoke can target human lungs directly and lead to progression of adenocarcinoma. To investigate physiological influence of lung cancer under long-term Cd exposure systematically, we established a chronic model by using A459 cells and provided a global view of genomic-/transcriptomic- regulations, which linked with our morphological observations (i.e., the cancer migration and invasion). Thus, our studies based on a long-term Cd exposure model not only deepened our understanding of Cd influence on adenocarcinoma, but also could be a useful indication for future therapeutic development.

## Materials and Methods

### Cell Culture and Treatment

Lung adenocarcinoma A549 cells were purchased from Cell Bank of Type Culture Collection of Chinese Academy of Sciences (Shanghai, China). A549 cells were cultured in DMEM medium (Invitrogen, Carlsbad, CA) that contained 10% fetal bovine serum, 100 U/ml penicillin, and 100 mg/ml streptomycin (Invitrogen). Cells were authenticated by short tandem repeat (STR) testing and amelogenin to the reference profile of A549 (ATCC CCL-185) (**Supplementary Figure 1**).

For cadmium exposures, cadmium chloride hemipentahydrate (Acros Organics, Gael, Belgium) was added to the media and applied evenly to the cultured cells. Cadmium chloride hemipentahydrate (Acros Organics, Gael, Belgium) at 1 μM was added to the culturing cells continually for 8 months to build the chronic low-dose Cd model.

### Examination of Cell Ultrastructure

Ultrastructure of A549+Cd cells and A549+H0 cells were observed using a transmission electron microscope (TEM). Cells were fixed with 2.5% glutaraldehyde (v/v) in 100 mM phosphate buffer (PBS, pH 7.0) for 2 h. Cells were washed three times with PBS and post-fixed in 1% osmium tetroxide (OsO_4_) for 1 h. Cells were dehydrated with an ethanol series, infiltrated, embedded in araldite and sectioned at 70 nm thickness using an ultramicrotome. Ultrathin sections were stained with 2% uranyl acetate and 0.2% lead citrate. Sections were examined using a TEM (JEOL JEM-1230 EX, Japan) at 80 Kv, and the images were observed.

### Immunofluorescent Staining

All fluorescent trackers were obtained from Beyotime Biotechnology (Shanghai, China). These trackers were used to stain responding intracellular proteins or organelles, which included cell actin (Actin-Tracker Green, at ratio of 1/40), mitochondria (Mito-Tracker Green, at ratio of 1/40), endoplasmic reticulum (ER-Tracker Red, at ratio of 1/40), Golgi (Golgi-Tracker Red, at ratio of 1/40), and lysosome (Lyso-Tracker Red, at ratio of 1/40), nucleus (DAPI, at 1 μg/mL). After being washed with PBS three times, cells were visualized under an inverted fluorescence microscope (Leica, Wetzlar, Germany).

### Scratch Test Migration Assay and Matrigel Transwell Invasive Assay

A two-chambered culture insert (Ibidi, Wisconsin, US) was placed in a 35 mm dish. The cells (2.5×10^5^ cells/chamber) were then seeded into the insert for 24 h until fully confluent attachment. After that, the inserts were removed using sterile forceps to create an even 500 μm cell-free gap. Cells were washed carefully with PBS once to remove any floating cells. Cell migration was analyzed at different time points (0 h, 6 h, 12 h, and 24 h) by a Nikon TMS-F phase contrast microscope (Tokyo, Japan).

For the Matrigel Transwell invasive assays, A549+Cd cells and A549+H0 cells were collected and resuspended at a density of 1x10^5^ in 100 ml of serum-free medium and then seeded into the upper chamber (Corning, USA) with a Matrigel-coated membrane (24-well insert; 8-mm pore size) according to the manufacturer’s protocol. Afterwards, we filled the lower chambers with DMEM that contained 10% FBS. After incubation for the indicated time, non-invasive cells on the upper surface of the upper chamber were removed mechanically by cotton swabs; the cells on the lower surface of filters were fixed with methanol for 30 min and stained with 0.1% crystal violet for 30 min. The number of invasive cells was counted in five random 200 fields using an inverted microscope (Olympus IX51; Olympus America Inc., Melville, NY, USA).

### WES Analysis and Exome Data Analyses

For exome sequencing, DNA of A549 cells (with/without Cd treatments) were extracted by a Qiagen genomic DNA extraction kit (Qiagen GmbH, Germany). DNA libraries were then prepared using a NEXTflexTM Rapid DNA Sequencing Kit (5144-02). The libraries were tested for enrichment by qPCR; their size distribution and concentration were determined by an Agilent Bioanalyzer 2100. After the library construction, WES was performed on an Illumina HiSeq3000 sequencer (version 3, Illumina, Inc., California, USA) in high-output mode with 150 bp paired-end reads. The consequent WES data were analyzed by a pipeline with three major steps: 1) quality control (QC) of raw data; 2) sequence read mapping; and 3) single-nucleotide polymorphism (SNP) analyses. In detail, QC was performed on the NGS raw data (FASTQ format) by using FastQC (38), which provides a thorough examination of the reads. Raw reads were cleaned by removal of adapter sequences and low-quality reads (Phred quality <20) and then applied to genome mapping. Different attributes of reads (e.g. Phred score, GC ratio, reads coverage, adapter/primer influences, and sequence duplicates) were checked, trimmed, and filtered to present high-quality reads for sequence mapping. The sequences were then mapped to the human genome (hg19) using the Burrows-Wheeler Aligner (0.7.12) (39). After mapping, GATK/Picard (40) software was employed to detect SNP loci with PHRED score >30 (i.e., error rate < 1/1000). Subsequently, ANNOVAR (41) was used to annotate SNP loci to determine whether the detected SNPs were in the known database (i.e., dbSNP138SNP database, 1000 Genome database and ESP6500 human exon database), gene functional annotation, exonic variant annotation, and heterozygosity.

GO and KEGG analysis were performed through the DAVID (https://david.ncifcrf.gov) database (42). Besides, to evaluate further the pathogenicity of these SNPs in 78 mutated genes associated with cell migration and invasion, we checked on Pathogenicity Calculator (http://calculator.clinicalgenome.org/site/cg-calculator) (43). To analyze the 78 mutated genes associated with cell migration and invasion, the human lung adenocarcinoma SNP mutation data and transcriptome data (normal: 54, tumor: 497) (TCGA-LUAD) were downloaded from the Cancer Genome Atlas (TCGA) database through DESeq2 installation package (44). The overlap of up-regulated mutated genes and down-regulated mutated genes were visualized using the R package “maftools”.

### RNA Sequencing and Data Analysis

Total RNA was extracted and purified by using Qiagen RNeasy Mini Kit (Hilden, Germany). The purity, concentration and integrity of total RNA were detected by NanoDrop 2000 spectrophotometer, Qubit 3.0 Fluorometer and Agilent 2100 Bioanalyzer, respectively. To retain all lncRNAs, which included those with or without a poly (A) tail, ribosomal RNA was removed by an Epicentre Ribo-zero™ rRNA Removal Kit (Epicentre, USA). The purified RNA was fragmented, converted into cDNA with adenylation of 3’ ends, and then amplified by using PCR. RNA-seq was then performed by Bai Hao Biological Technologies (Liaoning, China), using an Illumina HiSeq3000 platform (50 cycles with 150 base pair-end reads). Totals of 61,156,328 and 47,872,203 pair-end reads remain in A549+Cd and A549+H0 cells, respectively, after the QC process, which included the removal of adapter sequences, contaminated sequences, and low-quality sequences (Phred quality < 20 or N base > 10%). We also used the RNAcentral database (45) to remove the rRNAs that would affect the lncRNA data analysis, and 121,308,770 and 94,864,580 reads remained for A549+Cd and A549+H0 cells, respectively, for the mapping process. Tophat2 (46) was used to map reads to the Ensemble GRCh37/hg19 (iGenome version) reference with the default parameters (–read-mismatches = 2 and –read-gap-length = 2). Subsequently, we obtained 75,968,484 mapped reads that were distributed in exonic (80.9%), intergenic(3.4%), intronic(15.3%) and splicing(0.4%) in A549+Cd. A549+H0 had a similar result with 69,272,540 mapped reads distributed in exonic (78.6%), intergenic (4.0%), intronic (17.0%) and splicing (0.4%). The unique mapped reads were subjected to subsequent processing, such as removing PCR duplicates, before counting transcripts. Differential expression analysis (Cd treatments compared with Cd non-treatments) was conducted by a standard workflow, using AudicS (47) for genes, mRNA and lncRNA. The Benjamini–Hochberg multiple test correction was enabled by default during the analysis. All thresholds for significant differential expression were then set as q-value < 0.05 & |log2(Fold change)| > 1.5.

### Statistical Analysis

The results of Scratch test migration assay and Matrigel Transwell invasive assay were expressed as mean ± standard deviation calculated from three independent experiments. The data were analyzed with an independent samples t-test, SPSS 17.0 software. Values of *p* < 0.05 were taken as statistically significant. The workflow of DEseq2 and AudicS used the Benjamini–Hochberg multiple test correction (FDR) to correct *p*-value to *q*-value.

## Data Availability Statement

The datasets presented in this study can be found in online repositories. The names of the repository/repositories and accession number(s) can be found in the article/**Supplementary Material**.

## Author Contributions

SDD contributed to the conception of the work, designed the work and drafted the manuscript. SW participated in the acquisition and analysis of the data. JCZ and YNQ interpreted the data and revised the manuscript. All authors contributed to the article and approved the submitted version.

## Funding

This work was supported by grants from National Natural Science Foundation of China (No. 81401881 to SDD), Natural Science Foundation of Liaoning Province (No. 2014021018 to SDD), and Fundamental research program funding of Ninth People’s Hospital affiliated to Shanghai Jiao Tong university School of Medicine (No. JYZZ144 to SDD).

## Conflict of Interest

The authors declare that the research was conducted in the absence of any commercial or financial relationships that could be construed as a potential conflict of interest.

## Publisher’s Note

All claims expressed in this article are solely those of the authors and do not necessarily represent those of their affiliated organizations, or those of the publisher, the editors and the reviewers. Any product that may be evaluated in this article, or claim that may be made by its manufacturer, is not guaranteed or endorsed by the publisher.

## Supplementary Material

Supplementary Figure 1 STR profiles of parental A549 cells, A549+H0 cells and A549+Cd cells.All 30 STR loci detected in our cells were matched with the ATCC 30 STR loci of lung adenocarcinoma A549 cells, and the match degree was 100%.The result confirmed the identity of cells used in our study and excluded the possibility of cross-contamination during long-term cell passage.
