# Supplementary figures and images for: Multiomics Landscape Uncovers the Molecular Mechanism of the Malignant Evolution of Lung Adenocarcinoma Cells to Chronic Low Dose Cadmium Exposure
Source: Front Oncol. 2021 Nov 11;11:654687. doi: 10.3389/fonc.2021.654687 (PMC8631903; doi:10.3389/fonc.2021.654687)

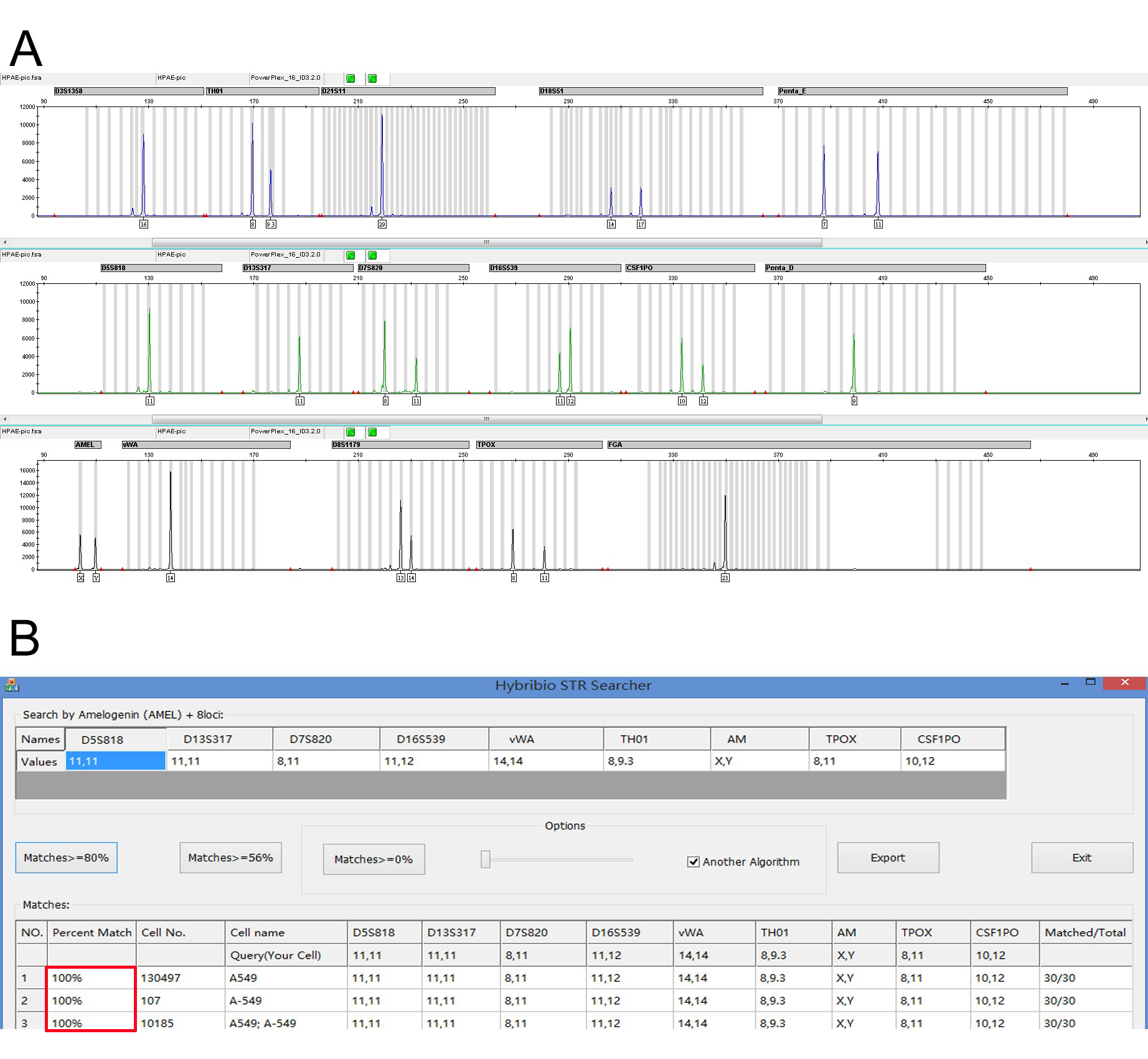

Supplement: Supplementary Figure 1 — STR profiles of parental A549 cells, A549+H0 cells and A549+Cd cells.All 30 STR loci detected in our cells were matched with the ATCC 30 STR loci of lung adenocarcinoma A549 cells, and the match degree was 100%.The result confirmed the identity of cells used in our study and excluded the possibility of cross-contamination during long-term cell passage. [file Image_1.jpeg]
