# Supplementary material for: Multiomics Landscape Uncovers the Molecular Mechanism of the Malignant Evolution of Lung Adenocarcinoma Cells to Chronic Low Dose Cadmium Exposure
Source: Front Oncol. 2021 Nov 11;11:654687. doi: 10.3389/fonc.2021.654687 (PMC8631903; doi:10.3389/fonc.2021.654687)
Supplement: Supplementary file 4 [file Table_3.docx]

**Supplemental Data 3** Unique SNPs associated with cell migration and invasion ability in A549+Cd cells

| **No.** | **Gene** | **Chrom** | **position** | **Change** | **Pathogenicity calculator** |
| --- | --- | --- | --- | --- | --- |
| 1 | OBSCN | chr1 | 228444565 | NM_001098623:exon4:c.A1505G:p.Q502R | undetermined |
|  |  |  |  | NM_001271223:exon4:c.A1505G:p.Q502R | undetermined |
|  |  |  |  | NM_052843:exon4:c.A1505G:p.Q502R | undetermined |
|  |  |  | 228494790 | NM_001098623:exon45:c.G12115A:p.G4039R | Undetermined |
|  |  |  |  | NM_052843:exon45:c.G12115A:p.G4039R | no |
|  |  |  |  | NM_001271223:exon56:c.G14986A:p.G4996R | no |
|  |  |  | 228509427 | NM_001098623:exon55:c.A14885G:p.D4962G | Undetermined |
|  |  |  |  | NM_052843:exon55:c.A14885G:p.D4962G | no |
|  |  |  |  | NM_001271223:exon66:c.A17756G:p.D5919G | no |
|  |  |  | 228528563 | NM_001098623:exon72:c.C17671G:p.Q5891E | Undetermined |
|  |  |  |  | NM_052843:exon72:c.C17671G:p.Q5891E | no |
|  |  |  |  | NM_001271223:exon83:c.C20542G:p.Q6848E | no |
|  |  |  | 228559994 | NM_001098623:exon94:c.C21515T:p.A7172V | Undetermined |
|  |  |  |  | NM_001271223:exon105:c.C24386T:p.A8129V | Undetermined |
| 2 | HRNR | chr1 | 152185762 | NM_001009931:exon3:c.G8343C:p.Q2781H | no |
|  |  |  | 152187183 | NM_001009931:exon3:c.G6922T:p.G2308C | Undetermined |
|  |  |  | 152187554 | NM_001009931:exon3:c.G6551A:p.S2184N | Undetermined |
|  |  |  | 152187562 | NM_001009931:exon3:c.T6543G:p.H2181Q | Undetermined |
|  |  |  | 152187935 | NM_001009931:exon3:c.G6170A:p.R2057Q | Undetermined |
|  |  |  | 152187945 | NM_001009931:exon3:c.G6160A:p.G2054S | Undetermined |
|  |  |  | 152188847 | NM_001009931:exon3:c.T5258C:p.V1753A | Undetermined |
|  |  |  | 152188920 | NM_001009931:exon3:c.G5185A:p.G1729S | Undetermined |
|  |  |  | 152191469 | NM_001009931:exon3:c.G2636A:p.G879D | no |
|  |  |  | 152191709 | NM_001009931:exon3:c.G2396C:p.S799T | Undetermined |
|  |  |  | 152192114 | NM_001009931:exon3:c.G1991A:p.R664Q | Undetermined |
|  |  |  | 152192163 | NM_001009931:exon3:c.C1942A:p.R648S | Undetermined |
|  |  |  | 152192631 | NM_001009931:exon3:c.G1474A:p.G492R | Undetermined |
|  |  |  | 152192687 | NM_001009931:exon3:c.A1418G:p.E473G | Undetermined |
|  |  |  | 152192825 | NM_001009931:exon3:c.G1280A:p.G427D | Undetermined |
|  |  |  | 152192978 | NM_001009931:exon3:c.A1127G:p.Q376R | Undetermined |
|  |  |  | 152193851 | NM_001009931:exon3:c.G254A:p.R85H | Undetermined |
| 3 | NID1 | chr1 | 236141174 | NM_002508:exon20:c.A3737G:p.Q1246R | Undetermined |
|  |  |  | 236208773 | NM_002508:exon3:c.G736A:p.V246I | Undetermined |
| 4 | PDE4DIP | chr1 | 144854581 | NM_001198834:exon42:c.A6889G:p.T2297A | no |
|  |  |  |  | NM_014644:exon42:c.A6889G:p.T2297A | no |
|  |  |  |  | NM_001198832:exon44:c.A6571G:p.T2191A | Undetermined |
|  |  |  | 144865850 | NM_001198834:exon35:c.C5730A:p.D1910E | no |
|  |  |  |  | NM_014644:exon35:c.C5730A:p.D1910E | no |
|  |  |  |  | NM_001198832:exon37:c.C5412A:p.D1804E | undetermined |
|  |  |  | 144866643 | NM_001198834:exon34:c.C5599T:p.R1867C | no |
|  |  |  |  | NM_014644:exon34:c.C5599T:p.R1867C | no |
|  |  |  |  | NM_001198832:exon36:c.C5281T:p.R1761C | undetermined |
|  |  |  | 144868170 | NM_001198834:exon33:c.G5269A:p.A1757T | no |
|  |  |  |  | NM_014644:exon33:c.G5269A:p.A1757T | no |
|  |  |  |  | NM_001198832:exon35:c.G4951A:p.A1651T | undetermined |
|  |  |  | 144871738 | NM_001198834:exon32:c.G5224T:p.A1742S | no |
|  |  |  |  | NM_014644:exon32:c.G5224T:p.A1742S | no |
|  |  |  | 144871755 | NM_001198834:exon32:c.T5207A:p.V1736E | no |
|  |  |  |  | NM_014644:exon32:c.T5207A:p.V1736E | no |
|  |  |  | 144871782 | NM_001009931:exon3:c.G6160A:p.G2054S | no |
|  |  |  |  | NM_014644:exon32:c.T5180C:p.L1727P | no |
|  |  |  | 144877176 | NM_001198834:exon28:c.G4511A:p.R1504Q | no |
|  |  |  |  | NM_014644:exon28:c.G4511A:p.R1504Q | no |
|  |  |  |  | NM_001198832:exon31:c.G4379A:p.R1460Q | undetermined |
|  |  |  | 144879264 | NM_001198834:exon27:c.T4186C:p.W1396R | no |
|  |  |  |  | NM_014644:exon27:c.T4186C:p.W1396R | no |
|  |  |  |  | NM_001198832:exon30:c.T4054C:p.W1352R | no |
|  |  |  | 144879375 | NM_001198834:exon27:c.A4075G:p.K1359E | no |
|  |  |  |  | NM_014644:exon27:c.A4075G:p.K1359E | no |
|  |  |  |  | NM_001198832:exon30:c.A3943G:p.K1315E | undetermined |
|  |  |  | 144880832 | NM_001198834:exon26:c.A3796G:p.K1266E | no |
|  |  |  |  | NM_014644:exon26:c.A3796G:p.K1266E | no |
|  |  |  |  | NM_001198832:exon29:c.A3664G:p.K1222E | undetermined |
|  |  |  | 144882823 | NM_001198834:exon24:c.G3196A:p.A1066T | no |
|  |  |  |  | NM_014644:exon24:c.G3196A:p.A1066T | no |
|  |  |  |  | NM_001198832:exon27:c.G3394A:p.A1132T | undetermined |
|  |  |  | 144886197 | NM_001198834:exon23:c.T3037A:p.F1013I | no |
|  |  |  |  | NM_014644:exon23:c.T3037A:p.F1013I | no |
|  |  |  |  | NM_001198832:exon26:c.T3235A:p.F1079I | undetermined |
|  |  |  | 144912153 | NM_001002811:exon11:c.T2611C:p.C871R | no |
|  |  |  |  | NM_001002812:exon15:c.T2122C:p.C708R | no |
|  |  |  |  | NM_001198834:exon15:c.T2122C:p.C708R | no |
|  |  |  |  | NM_014644:exon15:c.T2122C:p.C708R | no |
|  |  |  |  | NM_001198832:exon18:c.T2320C:p.C774R | no |
|  |  |  | 144912233 | NM_001002811:exon11:c.G2531A:p.R844H | undetermined |
|  |  |  |  | NM_001002812:exon15:c.G2042A:p.R681H | undetermined |
|  |  |  |  | NM_001198834:exon15:c.G2042A:p.R681H | no |
|  |  |  |  | NM_014644:exon15:c.G2042A:p.R681H | no |
|  |  |  |  | NM_001198832:exon18:c.G2240A:p.R747H | undetermined |
|  |  |  | 144916748 | NM_001002811:exon9:c.G2096C:p.S699T | undetermined |
|  |  |  |  | NM_001002812:exon13:c.G1607C:p.S536T | undetermined |
|  |  |  |  | NM_001198834:exon13:c.G1607C:p.S536T | no |
|  |  |  |  | NM_014644:exon13:c.G1607C:p.S536T | no |
|  |  |  |  | NM_001198832:exon16:c.G1805C:p.S602T | undetermined |
|  |  |  | 144918957 | NM_001002811:exon6:c.A1718T:p.E573V | undetermined |
|  |  |  |  | NM_001002812:exon10:c.A1229T:p.E410V | undetermined |
|  |  |  |  | NM_001198834:exon10:c.A1229T:p.E410V | no |
|  |  |  |  | NM_014644:exon10:c.A1229T:p.E410V | no |
|  |  |  |  | NM_001198832:exon13:c.A1427T:p.E476V | undetermined |
|  |  |  | 144922583 | NM_001002811:exon3:c.C1313T:p.S438L | undetermined |
|  |  |  |  | NM_001002812:exon7:c.C824T:p.S275L | undetermined |
|  |  |  |  | NM_001198834:exon7:c.C824T:p.S275L | no |
|  |  |  |  | NM_014644:exon7:c.C824T:p.S275L | no |
|  |  |  |  | NM_001198832:exon10:c.C1022T:p.S341L | undetermined |
|  |  |  | 144930940 | NM_001002811:exon1:c.A769G:p.K257E | undetermined |
|  |  |  | 144931330 | NM_001002811:exon1:c.G379A:p.A127T | undetermined |
|  |  |  | 144931392 | NM_001002811:exon1:c.C317T:p.A106V | undetermined |
|  |  |  | 144931461 | NM_001002811:exon1:c.T248A:p.L83Q | undetermined |
|  |  |  | 144994658 | NM_001002810:exon1:c.G74T:p.R25L | no |
|  |  |  |  | NM_001002812:exon1:c.G74T:p.R25L | undetermined |
|  |  |  |  | NM_001198834:exon1:c.G74T:p.R25L | no |
|  |  |  |  | NM_014644:exon1:c.G74T:p.R25L | no |
|  |  |  |  | NM_001195261:exon2:c.G83T:p.R28L | undetermined |
|  |  |  |  | NM_001198832:exon4:c.G272T:p.R91L | undetermined |
|  |  |  |  | NM_022359:exon4:c.G485T:p.R162L | no |
| 5 | TNN | chr1 | 175046789 | NM_022093:exon2:c.A235G:p.R79G | Undetermined |
|  |  |  | 175092674 | NM_022093:exon12:c.C2789T:p.P930L | undetermined |
|  |  |  | 175105996 | NM_022093:exon17:c.C3467T:p.A1156V | undetermined |
| 6 | TTN | chr2 | 179393691 | NM_003319:exon188:c.C79592T:p.T26531I | no |
|  |  |  |  | NM_133432:exon189:c.C79967T:p.T26656I | no |
|  |  |  |  | NM_133437:exon189:c.C80168T:p.T26723I | no |
|  |  |  |  | NM_133378:exon309:c.C99083T:p.T33028I | no |
|  |  |  |  | NM_001256850:exon310:c.C101864T:p.T33955I | no |
|  |  |  |  | NM_001267550:exon360:c.C106787T:p.T35596I | undetermined |
|  |  |  | 179397561 | NM_003319:exon186:c.G76586A:p.R25529H | no |
|  |  |  |  | NM_133432:exon187:c.G76961A:p.R25654H | no |
|  |  |  |  | NM_133437:exon187:c.G77162A:p.R25721H | no |
|  |  |  |  | NM_133378:exon307:c.G96077A:p.R32026H | no |
|  |  |  |  | NM_001256850:exon308:c.G98858A:p.R32953H | no |
|  |  |  |  | NM_001267550:exon358:c.G103781A:p.R34594H | undetermined |
|  |  |  | 179427536 | NM_003319:exon154:c.A56128G:p.I18710V | no |
|  |  |  |  | NM_133432:exon155:c.A56503G:p.I18835V | no |
|  |  |  |  | NM_133437:exon155:c.A56704G:p.I18902V | no |
|  |  |  |  | NM_133378:exon275:c.A75619G:p.I25207V | no |
|  |  |  |  | NM_001256850:exon276:c.A78400G:p.I26134V | no |
|  |  |  |  | NM_001267550:exon326:c.A83323G:p.I27775V | undetermined |
|  |  |  | 179430997 | NM_003319:exon154:c.C52667T:p.T17556M | no |
|  |  |  |  | NM_133432:exon155:c.C53042T:p.T17681M | no |
|  |  |  |  | NM_133437:exon155:c.C53243T:p.T17748M | no |
|  |  |  |  | NM_133378:exon275:c.C72158T:p.T24053M | no |
|  |  |  |  | NM_001256850:exon276:c.C74939T:p.T24980M | no |
|  |  |  |  | NM_001267550:exon326:c.C79862T:p.T26621M | no |
|  |  |  | 179432185 | NM_003319:exon154:c.T51479C:p.I17160T | no |
|  |  |  |  | NM_133432:exon155:c.T51854C:p.I17285T | no |
|  |  |  |  | NM_133437:exon155:c.T52055C:p.I17352T | no |
|  |  |  |  | NM_133378:exon275:c.T70970C:p.I23657T | no |
|  |  |  |  | NM_001256850:exon276:c.T73751C:p.I24584T | no |
|  |  |  |  | NM_001267550:exon326:c.T78674C:p.I26225T | undetermined |
|  |  |  | 179444768 | NM_003319:exon146:c.G40051C:p.A13351P | no |
|  |  |  |  | NM_133432:exon147:c.G40426C:p.A13476P | no |
|  |  |  |  | NM_133437:exon147:c.G40627C:p.A13543P | no |
|  |  |  |  | NM_133378:exon267:c.G59542C:p.A19848P | no |
|  |  |  |  | NM_001256850:exon268:c.G62323C:p.A20775P | no |
|  |  |  |  | NM_001267550:exon318:c.G67246C:p.A22416P | no |
|  |  |  | 179545859 | NM_133378:exon135:c.G29555A:p.R9852H | no |
|  |  |  |  | NM_001256850:exon136:c.G32336A:p.R10779H | no |
|  |  |  |  | NM_001267550:exon138:c.G33287A:p.R11096H | undetermined |
|  |  |  | 179554305 | NM_133378:exon120:c.G28132A:p.G9378R | no |
|  |  |  |  | NM_001256850:exon121:c.G30913A:p.G10305R | no |
|  |  |  |  | NM_001267550:exon123:c.G31864A:p.G10622R | undetermined |
|  |  |  | 179614411 | NM_133379:exon46:c.C12716T:p.A4239V | no |
|  |  |  | 179615887 | NM_133379:exon46:c.A11240G:p.D3747G | no |
|  |  |  | 179615931 | NM_133379:exon46:c.G11196C:p.L3732F | no |
|  |  |  | 179620951 | NM_133437:exon44:c.G10739A:p.G3580D | no |
|  |  |  |  | NM_001267550:exon46:c.G11252A:p.G3751D | undetermined |
|  |  |  | 179623758 | NM_003319:exon43:c.G10118A:p.S3373N | no |
|  |  |  |  | NM_133432:exon43:c.G10118A:p.S3373N | no |
|  |  |  |  | NM_133437:exon43:c.G10118A:p.S3373N | no |
|  |  |  |  | NM_001256850:exon44:c.G10256A:p.S3419N | no |
|  |  |  |  | NM_001267550:exon44:c.G10256A:p.S3419N | undetermined |
|  |  |  |  | NM_133378:exon44:c.G10256A:p.S3419N | no |
|  |  |  |  | NM_133379:exon44:c.G10256A:p.S3419N | no |
|  |  |  | 179629461 | NM_003319:exon41:c.G9643A:p.V3215M | no |
|  |  |  |  | NM_133432:exon41:c.G9643A:p.V3215M | no |
|  |  |  |  | NM_133437:exon41:c.G9643A:p.V3215M | no |
|  |  |  |  | NM_001256850:exon42:c.G9781A:p.V3261M | no |
|  |  |  |  | NM_001267550:exon42:c.G9781A:p.V3261M | undetermined |
|  |  |  |  | NM_133378:exon42:c.G9781A:p.V3261M | no |
|  |  |  |  | NM_133379:exon42:c.G9781A:p.V3261M | no |
| 7 | SPTBN1 | chr2 | 54870254 | NM_178313:exon18:c.C3954A:p.D1318E | Undetermined |
|  |  |  |  | NM_003128:exon19:c.C3993A:p.D1331E | undetermined |
| 8 | LRP2 | chr2 | 55494141 | NM_004525:exon69:c.A12628C:p.I4210L | Undetermined |
| 9 | ITSN2 | chr2 | 24439048 | NM_019595:exon31:c.T3779C:p.I1260T | Undetermined |
|  |  |  |  | NM_006277:exon32:c.T3860C:p.I1287T | Undetermined |
|  |  |  | 24524958 | NM_006277:exon10:c.G871A:p.V291I | Undetermined |
|  |  |  |  | NM_019595:exon10:c.G871A:p.V291I | Undetermined |
|  |  |  |  | NM_147152:exon10:c.G871A:p.V291I | Undetermined |
| 10 | MYO3B | chr2 | 171260787 | NM_001083615:exon20:c.G2308A:p.V770I | no |
|  |  |  |  | NM_138995:exon20:c.G2308A:p.V770I | no |
|  |  |  | 171260797 | NM_001083615:exon20:c.A2318G:p.E773G | no |
|  |  |  |  | NM_138995:exon20:c.A2318G:p.E773G | no |
|  |  |  | 171356274 | NM_001083615:exon27:c.G3245A:p.R1082K | no |
|  |  |  |  | NM_138995:exon27:c.G3245A:p.R1082K | no |
| 11 | ROCK2 | chr2 | 11359120 | NM_004850:exon10:c.C1292A:p.T431N | no |
| 12 | NEB | chr2 | 152352843 | NM_004543:exon140:c.G18829C:p.A6277P | no |
|  |  |  |  | NM_001164507:exon173:c.G24433C:p.A8145P | undetermined |
|  |  |  |  | NM_001164508:exon173:c.G24433C:p.A8145P | undetermined |
|  |  |  |  | NM_001271208:exon174:c.G24538C:p.A8180P | undetermined |
|  |  |  | 152404901 | NM_004543:exon103:c.C14939T:p.T4980I | no |
|  |  |  |  | NM_001164507:exon131:c.C20078T:p.T6693 | undetermined |
|  |  |  |  | NM_001164508:exon131:c.C20078T:p.T6693I | undetermined |
|  |  |  |  | NM_001271208:exon131:c.C20078T:p.T6693I | undetermined |
|  |  |  | 152422076 | NM_004543:exon88:c.G13166C:p.R4389T | no |
|  |  |  |  | NM_001164507:exon116:c.G18305C:p.R6102T | undetermined |
|  |  |  |  | NM_001164508:exon116:c.G18305C:p.R6102T | undetermined |
|  |  |  |  | NM_001271208:exon116:c.G18305C:p.R6102T | undetermined |
|  |  |  | 152436012 | NM_001164507:exon105:c.A16544C:p.K5515T | undetermined |
|  |  |  |  | NM_001164508:exon105:c.A16544C:p.K5515T | no |
|  |  |  |  | NM_001271208:exon105:c.A16544C:p.K5515T | no |
|  |  |  | 152460265 | NM_001164507:exon86:c.G13081A:p.E4361K | undetermined |
|  |  |  |  | NM_001164508:exon86:c.G13081A:p.E4361K | undetermined |
|  |  |  |  | NM_001271208:exon86:c.G13081A:p.E4361K | undetermined |
|  |  |  | 152471080 | NM_004543:exon73:c.C10546T:p.R3516C | undetermined |
|  |  |  |  | NM_001164507:exon77:c.C11311T:p.R3771C | undetermined |
|  |  |  |  | NM_001164508:exon77:c.C11311T:p.R3771C | undetermined |
|  |  |  |  | NM_001271208:exon77:c.C11311T:p.R3771C | undetermined |
|  |  |  | 152476028 | NM_004543:exon69:c.G10044C:p.W3348C | no |
|  |  |  |  | NM_001164507:exon73:c.G10809C:p.W3603C | undetermined |
|  |  |  |  | NM_001164508:exon73:c.G10809C:p.W3603C | undetermined |
|  |  |  |  | NM_001271208:exon73:c.G10809C:p.W3603C | undetermined |
|  |  |  | 152490458 | NM_001164507:exon65:c.T9124C:p.C3042R | undetermined |
|  |  |  |  | NM_001164508:exon65:c.T9124C:p.C3042R | undetermined |
|  |  |  |  | NM_001271208:exon65:c.T9124C:p.C3042R | undetermined |
|  |  |  | 152500449 | NM_001164507:exon57:c.G7839C:p.K2613N | undetermined |
|  |  |  |  | NM_001164508:exon57:c.G7839C:p.K2613N | undetermined |
|  |  |  |  | NM_001271208:exon57:c.G7839C:p.K2613N | undetermined |
|  |  |  |  | NM_004543:exon57:c.G7839C:p.K2613N | no |
|  |  |  | 152527572 | NM_001164507:exon38:c.G4471A:p.V1491M | undetermined |
|  |  |  |  | NM_001164508:exon38:c.G4471A:p.V1491M | undetermined |
|  |  |  |  | NM_001271208:exon38:c.G4471A:p.V1491M | undetermined |
|  |  |  |  | NM_004543:exon38:c.G4471A:p.V1491M | no |
|  |  |  | 152531077 | NM_001164507:exon36:c.T3901C:p.Y1301H | undetermined |
|  |  |  |  | NM_001164508:exon36:c.T3901C:p.Y1301H | undetermined |
|  |  |  |  | NM_001271208:exon36:c.T3901C:p.Y1301H | undetermined |
|  |  |  |  | NM_004543:exon36:c.T3901C:p.Y1301H | no |
|  |  |  | 152536498 | NM_001164507:exon31:c.A3081T:p.K1027N | undetermined |
|  |  |  |  | NM_001164508:exon31:c.A3081T:p.K1027N | undetermined |
|  |  |  |  | NM_001271208:exon31:c.A3081T:p.K1027N | undetermined |
|  |  |  |  | NM_004543:exon31:c.A3081T:p.K1027N | no |
| 13 | DNAH6 | chr2 | 84924823 | NM_001370:exon47:c.T7649C:p.V2550A | undetermined |
| 14 | CKAP2L | chr2 | 113498566 | NM_152515:exon8:c.T1841C:p.L614S | no |
|  |  |  | 113513825 | NM_152515:exon4:c.A1123G:p.I375V | no |
| 15 | MYO7B | chr2 | 128321770 | NM_001080527:exon3:c.G61A:p.G21S | undetermined |
|  |  |  | 128394955 | NM_001080527:exon47:c.A6314G:p.Q2105R | undetermined |
| 16 | TMEM214 | chr2 | 27260469 | NM_001083590:exon8:c.G916A:p.V306M | undetermined |
|  |  |  |  | NM_017727:exon9:c.G1051A:p.V351M | no |
| 17 | ASTL | chr2 | 96795608 | NM_001002036:exon8:c.A829C:p.K277Q | no |
|  |  |  | 96795857 | NM_001002036:exon7:c.A665G:p.Q222R | no |
| 18 | ITGAV | chr2 | 187532417 | NM_001145000:exon22:c.G2239A:p.V747I | no |
|  |  |  |  | NM_001144999:exon24:c.G2209A:p.V737I | no |
|  |  |  |  | NM_002210:exon24:c.G2347A:p.V783I | no |
| 19 | SERPINI1 | chr3 | 167506937 | NM_001122752:exon2:c.C21G:p.F7L | undetermined |
|  |  |  |  | NM_005025:exon2:c.C21G:p.F7L | no |
| 20 | MUC4 | chr3 | 195489009 | NM_138297:exon12:c.G1600T:p.A534S | no |
|  |  |  |  | NM_004532:exon13:c.G1753T:p.A585S | no |
|  |  |  |  | NM_018406:exon14:c.G14461T:p.A4821S | no |
|  |  |  | 195495916 | NM_138297:exon5:c.C645G:p.N215K | no |
|  |  |  |  | NM_004532:exon6:c.C798G:p.N266K | no |
|  |  |  |  | NM_018406:exon7:c.C13506G:p.N4502K | no |
|  |  |  | 195497174 | NM_138297:exon4:c.G450C:p.M150I | no |
|  |  |  |  | NM_004532:exon5:c.G603C:p.M201I | no |
|  |  |  |  | NM_018406:exon6:c.G13311C:p.M4437I | no |
|  |  |  | 195501149 | NM_138297:exon2:c.G110A:p.G37D | no |
|  |  |  |  | NM_004532:exon3:c.G263A:p.G88D | no |
|  |  |  |  | NM_018406:exon4:c.G12971A:p.G4324D | no |
|  |  |  | 195505664 | NM_018406:exon2:c.C12787T:p.P4263S | no |
|  |  |  | 195505788 | NM_018406:exon2:c.C12663G:p.H4221Q | no |
|  |  |  | 195505801 | NM_018406:exon2:c.C12650T:p.A4217V | no |
|  |  |  | 195505814 | NM_018406:exon2:c.G12637A:p.D4213N | no |
|  |  |  | 195505907 | NM_018406:exon2:c.A12544C:p.T4182P | no |
|  |  |  | 195505930 | NM_018406:exon2:c.G12521C:p.G4174A | no |
|  |  |  | 195506099 | NM_018406:exon2:c.A12352G:p.T4118A | no |
|  |  |  | 195506354 | NM_018406:exon2:c.G12097C:p.V4033L | no |
|  |  |  | 195506569 | NM_018406:exon2:c.T11882C:p.V3961A | no |
|  |  |  | 195506746 | NM_018406:exon2:c.C11705T:p.A3902V | no |
|  |  |  | 195506753 | NM_018406:exon2:c.C11698G:p.R3900G | no |
|  |  |  | 195506940 | NM_018406:exon2:c.C11511G:p.H3837Q | no |
|  |  |  | 195506953 | NM_018406:exon2:c.C11498T:p.A3833V | no |
|  |  |  | 195506974 | NM_018406:exon2:c.C11477T:p.P3826L | no |
|  |  |  | 195507001 | NM_018406:exon2:c.T11450C:p.V3817A | no |
|  |  |  | 195507010 | NM_018406:exon2:c.T11441C:p.L3814P | no |
|  |  |  | 195507062 | NM_018406:exon2:c.G11389A:p.D3797N | no |
|  |  |  | 195507107 | NM_018406:exon2:c.G11344A:p.A3782T | no |
|  |  |  | 195507166 | NM_018406:exon2:c.T11285C:p.L3762P | no |
|  |  |  | 195507193 | NM_018406:exon2:c.C11258T:p.A3753V | no |
|  |  |  | 195507226 | NM_018406:exon2:c.T11225C:p.V3742A | no |
|  |  |  | 195507242 | NM_018406:exon2:c.G11209T:p.A3737S | no |
|  |  |  | 195507262 | NM_018406:exon2:c.A11189C:p.H3730P | no |
|  |  |  | 195507323 | NM_018406:exon2:c.A11128G:p.T3710A | no |
|  |  |  | 195507332 | NM_018406:exon2:c.T11119A:p.S3707T | no |
|  |  |  | 195507349 | NM_018406:exon2:c.T11102G:p.I3701S | no |
|  |  |  | 195507365 | NM_018406:exon2:c.C11086T:p.P3696S | no |
|  |  |  | 195507372 | NM_018406:exon2:c.G11079C:p.Q3693H | no |
|  |  |  | 195507379 | NM_018406:exon2:c.C11072G:p.T3691R | no |
|  |  |  | 195507385 | NM_018406:exon2:c.C11066T:p.A3689V | no |
|  |  |  | 195507406 | NM_018406:exon2:c.C11045T:p.P3682L | no |
|  |  |  | 195507412 | NM_018406:exon2:c.G11039C:p.R3680P | no |
|  |  |  | 195507433 | NM_018406:exon2:c.C11018T:p.A3673V | no |
|  |  |  | 195507461 | NM_018406:exon2:c.C10990T:p.P3664S | no |
|  |  |  | 195507502 | NM_018406:exon2:c.T10949C:p.L3650P | no |
|  |  |  | 195507518 | NM_018406:exon2:c.G10933C:p.D3645H | no |
|  |  |  | 195507564 | NM_018406:exon2:c.G10887C:p.Q3629H | no |
|  |  |  | 195507604 | NM_018406:exon2:c.G10847C:p.R3616P | no |
|  |  |  | 195507683 | NM_018406:exon2:c.G10768A:p.A3590T | no |
|  |  |  | 195507694 | NM_018406:exon2:c.T10757C:p.L3586P | no |
|  |  |  | 195507731 | NM_018406:exon2:c.C10720T:p.L3574F | no |
|  |  |  | 195507756 | NM_018406:exon2:c.G10695C:p.Q3565H | no |
|  |  |  | 195507779 | NM_018406:exon2:c.G10672A:p.A3558T | no |
|  |  |  | 195507827 | NM_018406:exon2:c.C10624T:p.L3542F | no |
|  |  |  | 195508046 | NM_018406:exon2:c.G10405C:p.D3469H | no |
|  |  |  | 195508091 | NM_018406:exon2:c.A10360G:p.T3454A | no |
|  |  |  | 195508108 | NM_018406:exon2:c.C10343T:p.S3448L | no |
|  |  |  | 195508130 | NM_018406:exon2:c.G10321C:p.V3441L | no |
|  |  |  | 195508462 | NM_018406:exon2:c.A9989C:p.H3330P | no |
|  |  |  | 195508510 | NM_018406:exon2:c.T9941C:p.L3314P | no |
|  |  |  | 195508558 | NM_018406:exon2:c.T9893C:p.L3298P | no |
|  |  |  | 195508661 | NM_018406:exon2:c.T9790C:p.S3264P | no |
|  |  |  | 195508846 | NM_018406:exon2:c.T9605C:p.L3202P | no |
|  |  |  | 195508956 | NM_018406:exon2:c.G9495C:p.Q3165H | no |
|  |  |  | 195509045 | NM_018406:exon2:c.G9406C:p.A3136P | no |
|  |  |  | 195509278 | NM_018406:exon2:c.A9173C:p.H3058P | no |
|  |  |  | 195509287 | NM_018406:exon2:c.A9164C:p.N3055T | no |
|  |  |  | 195509443 | NM_018406:exon2:c.T9008C:p.I3003T | no |
|  |  |  | 195509497 | NM_018406:exon2:c.C8954T:p.A2985V | no |
|  |  |  | 195509515 | NM_018406:exon2:c.A8936T:p.D2979V | no |
|  |  |  | 195509573 | NM_018406:exon2:c.T8878C:p.S2960P | no |
|  |  |  | 195509651 | NM_018406:exon2:c.C8800T:p.L2934F | no |
|  |  |  | 195509676 | NM_018406:exon2:c.G8775C:p.Q2925H | no |
|  |  |  | 195509689 | NM_018406:exon2:c.C8762T:p.A2921V | no |
|  |  |  | 195509795 | NM_018406:exon2:c.G8656A:p.A2886T | no |
|  |  |  | 195509861 | NM_018406:exon2:c.T8590C:p.S2864P | no |
|  |  |  | 195509974 | NM_018406:exon2:c.T8477C:p.F2826S | no |
|  |  |  | 195510094 | NM_018406:exon2:c.A8357C:p.H2786P | no |
|  |  |  | 195510217 | NM_018406:exon2:c.T8234C:p.V2745A | no |
|  |  |  | 195510228 | NM_018406:exon2:c.G8223C:p.E2741D | no |
|  |  |  | 195510238 | NM_018406:exon2:c.T8213C:p.L2738P | no |
|  |  |  | 195510293 | NM_018406:exon2:c.T8158C:p.S2720P | no |
|  |  |  | 195510310 | NM_018406:exon2:c.A8141C:p.Y2714S | no |
|  |  |  | 195510341 | NM_018406:exon2:c.T8110C:p.S2704P | no |
|  |  |  | 195510389 | NM_018406:exon2:c.T8062C:p.S2688P | no |
|  |  |  | 195510396 | NM_018406:exon2:c.T8055G:p.H2685Q | no |
|  |  |  | 195510526 | NM_018406:exon2:c.T7925C:p.L2642P | no |
|  |  |  | 195510636 | NM_018406:exon2:c.G7815C:p.Q2605H | no |
|  |  |  | 195510655 | NM_018406:exon2:c.A7796C:p.Y2599S | no |
|  |  |  | 195510686 | NM_018406:exon2:c.G7765C:p.D2589H | no |
|  |  |  | 195510707 | NM_018406:exon2:c.A7744C:p.T2582P | no |
|  |  |  | 195510718 | NM_018406:exon2:c.C7733A:p.P2578H | no |
|  |  |  | 195510773 | NM_018406:exon2:c.T7678C:p.S2560P | no |
|  |  |  | 195510827 | NM_018406:exon2:c.G7624A:p.A2542T | no |
|  |  |  | 195511142 | NM_018406:exon2:c.A7309G:p.N2437D | no |
|  |  |  | 195511331 | NM_018406:exon2:c.T7120C:p.S2374P | no |
|  |  |  | 195511369 | NM_018406:exon2:c.T7082C:p.V2361A | no |
|  |  |  | 195511390 | NM_018406:exon2:c.A7061C:p.H2354P | no |
|  |  |  | 195511403 | NM_018406:exon2:c.G7048A:p.A2350T | no |
|  |  |  | 195511412 | NM_018406:exon2:c.A7039T:p.T2347S | no |
|  |  |  | 195511454 | NM_018406:exon2:c.G6997C:p.D2333H | no |
|  |  |  | 195511474 | NM_018406:exon2:c.T6977C:p.L2326P | no |
|  |  |  | 195511513 | NM_018406:exon2:c.C6938T:p.A2313V | no |
|  |  |  | 195511534 | NM_018406:exon2:c.A6917C:p.H2306P | no |
|  |  |  | 195511547 | NM_018406:exon2:c.G6904A:p.A2302T | no |
|  |  |  | 195511556 | NM_018406:exon2:c.A6895T:p.T2299S | no |
|  |  |  | 195511780 | NM_018406:exon2:c.C6671T:p.P2224L | no |
|  |  |  | 195511814 | NM_018406:exon2:c.A6637G:p.S2213G | no |
|  |  |  | 195511836 | NM_018406:exon2:c.G6615C:p.Q2205H | no |
|  |  |  | 195511877 | NM_018406:exon2:c.C6574T:p.P2192S | no |
|  |  |  | 195511925 | NM_018406:exon2:c.T6526C:p.S2176P | no |
|  |  |  | 195511945 | NM_018406:exon2:c.C6506T:p.A2169V | no |
|  |  |  | 195511993 | NM_018406:exon2:c.T6458C:p.V2153A | no |
|  |  |  | 195512004 | NM_018406:exon2:c.A6447C:p.E2149D | no |
|  |  |  | 195512042 | NM_018406:exon2:c.A6409G:p.T2137A | no |
|  |  |  | 195512107 | NM_018406:exon2:c.A6344T:p.D2115V | no |
|  |  |  | 195512186 | NM_018406:exon2:c.A6265G:p.I2089V | no |
|  |  |  | 195512212 | NM_018406:exon2:c.C6239A:p.P2080H | no |
|  |  |  | 195512245 | NM_018406:exon2:c.A6206G:p.N2069S | no |
|  |  |  | 195512287 | NM_018406:exon2:c.C6164T:p.S2055F | no |
|  |  |  | 195512597 | NM_018406:exon2:c.C5854T:p.P1952S | no |
|  |  |  | 195513180 | NM_018406:exon2:c.G5271C:p.Q1757H | no |
|  |  |  | 195513433 | NM_018406:exon2:c.C5018T:p.A1673V | no |
|  |  |  | 195513491 | NM_018406:exon2:c.C4960A:p.P1654T | no |
|  |  |  | 195513502 | NM_018406:exon2:c.A4949C:p.H1650P | no |
|  |  |  | 195513566 | NM_018406:exon2:c.G4885C:p.D1629H | no |
|  |  |  | 195513667 | NM_018406:exon2:c.A4784C:p.K1595T | no |
|  |  |  | 195513680 | NM_018406:exon2:c.T4771A:p.S1591T | no |
|  |  |  | 195513779 | NM_018406:exon2:c.G4672A:p.A1558T | no |
|  |  |  | 195514846 | NM_018406:exon2:c.T3605C:p.L1202P | no |
|  |  |  | 195514948 | NM_018406:exon2:c.C3503T:p.P1168L | no |
|  |  |  | 195515017 | NM_018406:exon2:c.T3434C:p.V1145A | no |
|  |  |  | 195515045 | NM_018406:exon2:c.T3406C:p.S1136P | no |
|  |  |  | 195515141 | NM_018406:exon2:c.T3310C:p.S1104P | no |
|  |  |  | 195515194 | NM_018406:exon2:c.C3257G:p.T1086S | no |
|  |  |  | 195515290 | NM_018406:exon2:c.G3161C:p.S1054T | no |
|  |  |  | 195515315 | NM_018406:exon2:c.T3136C:p.F1046L | no |
|  |  |  | 195515387 | NM_018406:exon2:c.A3064G:p.T1022A | no |
|  |  |  | 195515401 | NM_018406:exon2:c.T3050A:p.V1017E | no |
|  |  |  | 195515449 | NM_018406:exon2:c.T3002A:p.V1001E | no |
|  |  |  | 195515470 | NM_018406:exon2:c.A2981C:p.H994P | no |
|  |  |  | 195515594 | NM_018406:exon2:c.G2857C:p.E953Q | no |
|  |  |  | 195515617 | NM_018406:exon2:c.C2834G:p.T945S | no |
|  |  |  | 195516878 | NM_018406:exon2:c.A1573G:p.T525A | no |
|  |  |  | 195517258 | NM_018406:exon2:c.C1193T:p.T398I | no |
|  |  |  | 195517321 | NM_018406:exon2:c.C1130T:p.T377I | no |
|  |  |  | 195517553 | NM_018406:exon2:c.T898G:p.F300V | no |
|  |  |  | 195518330 | NM_018406:exon2:c.G121C:p.A41P | no |
| 21 | FAT1 | chr4 | 187540040 | NM_005245:exon10:c.G7700A:p.R2567H | no |
|  |  |  | 187557893 | NM_005245:exon5:c.A3818G:p.H1273R | no |
| 22 | PCDHA4 | chr5 | 140186937 | NM_018907:exon1:c.G165T:p.E55D | no |
|  |  |  |  | NM_031500:exon1:c.G165T:p.E55D | undetermined |
|  |  |  | 140186990 | NM_018907:exon1:c.G218A:p.G73D | no |
|  |  |  |  | NM_031500:exon1:c.G218A:p.G73D | undetermined |
|  |  |  | 140187322 | NM_018907:exon1:c.C550T:p.P184S | no |
|  |  |  |  | NM_031500:exon1:c.C550T:p.P184S | undetermined |
| 23 | MYO10 | chr5 | 16711087 | NM_012334:exon21:c.G2099A:p.R700Q | undetermined |
|  |  |  | 16769273 | NM_012334:exon10:c.C970T:p.R324W | undetermined |
| 24 | PCDHA8 | chr5 | 140221139 | NM_018911:exon1:c.G233A:p.S78N | undetermined |
|  |  |  |  | NM_031856:exon1:c.G233A:p.S78N | undetermined |
|  |  |  | 140221195 | NM_018911:exon1:c.G289C:p.G97R | undetermined |
|  |  |  |  | NM_031856:exon1:c.G289C:p.G97R | undetermined |
|  |  |  | 140222157 | NM_018911:exon1:c.A1251C:p.R417S | undetermined |
|  |  |  |  | NM_031856:exon1:c.A1251C:p.R417S | undetermined |
|  |  |  | 140222641 | NM_018911:exon1:c.A1735G:p.K579E | undetermined |
|  |  |  |  | NM_031856:exon1:c.A1735G:p.K579E | undetermined |
|  |  |  | 140222738 | NM_018911:exon1:c.T1832A:p.L611Q | undetermined |
|  |  |  |  | NM_031856:exon1:c.T1832A:p.L611Q | undetermined |
| 25 | GPR98 | chr5 | 89943571 | NM_032119:exon17:c.G3279T:p.L1093F | no |
|  |  |  | 89979589 | NM_032119:exon28:c.G5851A:p.V1951I | no |
|  |  |  | 89979698 | NM_032119:exon28:c.C5960T:p.P1987L | no |
|  |  |  | 89985882 | NM_032119:exon30:c.A6695G:p.Y2232C | no |
|  |  |  | 89988504 | NM_032119:exon32:c.A7034G:p.N2345S | no |
|  |  |  | 89990324 | NM_032119:exon33:c.A7751G:p.N2584S | no |
|  |  |  | 90024735 | NM_032119:exon49:c.G10411A:p.E3471K | no |
|  |  |  | 90052289 | NM_032119:exon56:c.G11599A:p.E3867K | no |
|  |  |  | 90119324 | NM_032119:exon76:c.G16279A:p.V5427M | no |
| 26 | PCDHGC5 | chr5 | 140870516 | NM_018929:exon1:c.A1709G:p.D570G | undetermined |
|  |  |  |  | NM_032407:exon1:c.A1709G:p.D570G | no |
| 27 | PCDHB12 | chr5 | 140590393 | NM_018932:exon1:c.C1914G:p.H638Q | no |
|  |  |  | 140590766 | NM_018932:exon1:c.A2287G:p.K763E | no |
| 28 | PCDHGA7 | chr5 | 140763029 | NM_018920:exon1:c.A563G:p.E188G | no |
|  |  |  |  | NM_032087:exon1:c.A563G:p.E188G | undetermined |
|  |  |  | 140764394 | NM_018920:exon1:c.T1928G:p.V643G | no |
|  |  |  |  | NM_032087:exon1:c.T1928G:p.V643G | undetermined |
| 29 | PCDHB8 | chr5 | 140558528 | NM_019120:exon1:c.A913G:p.K305E | no |
|  |  |  | 140559320 | NM_019120:exon1:c.G1705A:p.G569S | no |
|  |  |  | 140559532 | NM_019120:exon1:c.G1917C:p.Q639H | no |
|  |  |  | 140559596 | NM_019120:exon1:c.T1981G:p.L661V | no |
|  |  |  | 140559849 | NM_019120:exon1:c.G2234A:p.S745N | no |
|  |  |  | 140559914 | NM_019120:exon1:c.T2299C:p.F767L | no |
| 30 | CAST | chr5 | 96086334 | NM_173060:exon17:c.G1157C:p.C386S | no |
|  |  |  |  | NM_001042440:exon18:c.G1349C:p.C450S | no |
|  |  |  |  | NM_001190442:exon19:c.G1184C:p.C395S | undetermined |
| 31 | MAP1B | chr5 | 71490962 | NM_005909:exon5:c.A1780G:p.I594V | no |
| 32 | DST | chr6 | 56417282 | NM_015548:exon42:c.G8439A:p.M2813I | no |
|  |  |  | 56417545 | NM_015548:exon42:c.A8176G:p.T2726A | no |
|  |  |  | 56420158 | NM_015548:exon41:c.G7252A:p.V2418I | no |
|  |  |  | 56480500 | NM_001723:exon24:c.A7765G:p.I2589V | no |
| 33 | RPS6KA2 | chr6 | 166862258 | NM_021135:exon14:c.G1286C:p.C429S | no |
|  |  |  |  | NM_001006932:exon15:c.G1310C:p.C437S | undetermined |
| 34 | AEBP1 | chr7 | 44147485 | NM_001129:exon5:c.C817A:p.P273T | no |
|  |  |  | 44153780 | NM_001129:exon21:c.A3397G:p.K1133E | no |
|  |  |  | 44153825 | NM_001129:exon21:c.G3442A:p.V1148I | no |
| 35 | PCLO | chr7 | 82582846 | NM_014510:exon5:c.G7423A:p.V2475I | undetermined |
|  |  |  |  | NM_033026:exon5:c.G7423A:p.V2475I | no |
|  |  |  | 82764425 | NM_014510:exon3:c.G2441C:p.S814T | undetermined |
|  |  |  |  | NM_033026:exon3:c.G2441C:p.S814T | no |
| 36 | RADIL | chr7 | 4841470 | NM_018059:exon12:c.A2656G:p.S886G | no |
| 37 | TRIP6 | chr7 | 100465824 | NM_003302:exon3:c.G332A:p.R111Q | undetermined |
| 38 | SSPO | chr7 | 149474393 | NM_198455:exon4:c.A437G:p.Q146R | undetermined |
|  |  |  | 149480638 | NM_198455:exon17:c.C2344T:p.R782C | undetermined |
|  |  |  | 149483237 | NM_198455:exon23:c.G3305A:p.G1102E | undetermined |
|  |  |  | 149485059 | NM_198455:exon26:c.T3814C:p.S1272P | undetermined |
| 39 | ANXA13 | chr8 | 124710729 | NM_004306:exon4:c.G257A:p.R86H | undetermined |
|  |  |  |  | NM_001003954:exon5:c.G380A:p.R127H | undetermined |
| 40 | GSDMC | chr8 | 130760850 | NM_031415:exon14:c.T1424C:p.M475T | undetermined |
|  |  |  | 130789698 | NM_031415:exon2:c.C136T:p.R46C | undetermined |
| 41 | PLEC | chr8 | 144995494 | NM_201378:exon32:c.G8453A:p.R2818H | no |
|  |  |  |  | NM_201379:exon32:c.G8429A:p.R2810H | undetermined |
|  |  |  |  | NM_201380:exon32:c.G8906A:p.R2969H | no |
|  |  |  |  | NM_201381:exon32:c.G8399A:p.R2800H | undetermined |
|  |  |  |  | NM_201382:exon32:c.G8495A:p.R2832H | no |
|  |  |  |  | NM_201383:exon32:c.G8507A:p.R2836H | undetermined |
|  |  |  |  | NM_201384:exon32:c.G8495A:p.R2832H | undetermined |
|  |  |  |  | NM_000445:exon33:c.G8576A:p.R2859H | no |
|  |  |  | 144996029 | NM_201378:exon32:c.T7918C:p.S2640P | no |
|  |  |  |  | NM_201379:exon32:c.T7894C:p.S2632P | undetermined |
|  |  |  |  | NM_201380:exon32:c.T8371C:p.S2791P | no |
|  |  |  |  | NM_201381:exon32:c.T7864C:p.S2622P | undetermined |
|  |  |  |  | NM_201382:exon32:c.T7960C:p.S2654P | no |
|  |  |  |  | NM_201383:exon32:c.T7972C:p.S2658P | undetermined |
|  |  |  |  | NM_201384:exon32:c.T7960C:p.S2654P | undetermined |
|  |  |  |  | NM_000445:exon33:c.T8041C:p.S2681P | no |
|  |  |  | 145001031 | NM_201378:exon30:c.A3923G:p.H1308R | no |
|  |  |  |  | NM_201379:exon30:c.A3899G:p.H1300R | no |
|  |  |  |  | NM_201380:exon30:c.A4376G:p.H1459R | no |
|  |  |  |  | NM_201381:exon30:c.A3869G:p.H1290R | undetermined |
|  |  |  |  | NM_201382:exon30:c.A3965G:p.H1322R | no |
|  |  |  |  | NM_201383:exon30:c.A3977G:p.H1326R | undetermined |
|  |  |  |  | NM_201384:exon30:c.A3965G:p.H1322R | undetermined |
|  |  |  |  | NM_000445:exon31:c.A4046G:p.H1349R | no |
|  |  |  | 145001588 | NM_201378:exon27:c.G3704A:p.R1235Q | no |
|  |  |  |  | NM_201379:exon27:c.G3680A:p.R1227Q | undetermined |
|  |  |  |  | NM_201380:exon27:c.G4157A:p.R1386Q | no |
|  |  |  |  | NM_201381:exon27:c.G3650A:p.R1217Q | undetermined |
|  |  |  |  | NM_201382:exon27:c.G3746A:p.R1249Q | no |
|  |  |  |  | NM_201383:exon27:c.G3758A:p.R1253Q | undetermined |
|  |  |  |  | NM_201384:exon27:c.G3746A:p.R1249Q | undetermined |
|  |  |  |  | NM_000445:exon28:c.G3827A:p.R1276Q | no |
|  |  |  | 145007187 | NM_201378:exon14:c.C1469T:p.A490V | no |
|  |  |  |  | NM_201379:exon14:c.C1445T:p.A482V | undetermined |
|  |  |  |  | NM_201380:exon14:c.C1922T:p.A641V | no |
|  |  |  |  | NM_201381:exon14:c.C1415T:p.A472V | undetermined |
|  |  |  |  | NM_201382:exon14:c.C1511T:p.A504V | no |
|  |  |  |  | NM_201383:exon14:c.C1523T:p.A508V | undetermined |
|  |  |  |  | NM_201384:exon14:c.C1511T:p.A504V | undetermined |
|  |  |  |  | NM_000445:exon15:c.C1592T:p.A531V | no |
| 42 | MCPH1 | chr8 | 6296550 | NM_001172574:exon6:c.G513T:p.R171S | undetermined |
|  |  |  |  | NM_024596:exon6:c.G513T:p.R171S | no |
|  |  |  | 6302183 | NM_001172575:exon7:c.G796C:p.D266H | undetermined |
|  |  |  |  | NM_001172574:exon8:c.G940C:p.D314H | undetermined |
|  |  |  |  | NM_024596:exon8:c.G940C:p.D314H | no |
|  |  |  | 6302418 | NM_001172575:exon7:c.A1031G:p.D344G | undetermined |
|  |  |  |  | NM_001172574:exon8:c.A1175G:p.D392G | undetermined |
|  |  |  |  | NM_024596:exon8:c.A1175G:p.D392G | no |
|  |  |  | 6479042 | NM_024596:exon13:c.C2282T:p.A761V | no |
|  |  |  |  | NM_024596:exon13:c.C2282T:p.A761V | no |
| 43 | KIAA0368 | chr9 | 114174415 | NM_001080398:exon22:c.A2627G:p.N876S | no |
| 44 | LAMC3 | chr9 | 133884820 | NM_006059:exon1:c.T219G:p.H73Q | no |
|  |  |  | 133962930 | NM_006059:exon26:c.C4298T:p.T1433M | no |
| 45 | MMP27 | chr11 | 102565820 | NM_022122:exon7:c.G911T:p.W304L | undetermined |
|  |  |  | 102567207 | NM_022122:exon6:c.A797T:p.E266V | undetermined |
| 46 | MMP8 | chr11 | 102593248 | NM_002424:exon2:c.A259G:p.K87E | undetermined |
| 47 | CD248 | chr11 | 66083129 | NM_020404:exon1:c.A1370G:p.H457R | undetermined |
| 48 | KRT3 | chr12 | 53186088 | NM_057088:exon5:c.C1123G:p.R375G | undetermined |
| 49 | DNAH10 | chr12 | 124265687 | NM_207437:exon6:c.T499C:p.S167P | no |
|  |  |  | 124274474 | NM_207437:exon11:c.A1438G:p.I480V | no |
|  |  |  | 124325977 | NM_207437:exon29:c.T4891G:p.L1631V | no |
|  |  |  | 124330311 | NM_207437:exon30:c.C5171T:p.T1724M | no |
| 50 | RYR3 | chr15 | 33905410 | NM_001036:exon19:c.A2191G:p.I731V | undetermined |
|  |  |  |  | NM_001243996:exon19:c.A2191G:p.I731V | undetermined |
|  |  |  | 33954652 | NM_001036:exon35:c.C4921T:p.R1641C | no |
|  |  |  |  | NM_001243996:exon35:c.C4921T:p.R1641C | undetermined |
|  |  |  | 34016274 | NM_001036:exon45:c.G6809A:p.G2270E | no |
|  |  |  |  | NM_001243996:exon45:c.G6809A:p.G2270E | undetermined |
| 51 | MYO5A | chr15 | 52689631 | MYO5A:NM_000259:exon10:c.A1086C:p.E362D | no |
|  |  |  |  | NM_001142495:exon10:c.A1086C:p.E362D | undetermined |
| 52 | MNS1 | chr15 | 56756285 | NM_018365:exon2:c.A164C:p.Q55P | undetermined |
| 53 | MAP2K5 | chr15 | 67984889 | NM_001206804:exon14:c.A812C:p.Q271P | undetermined |
|  |  |  |  | NM_002757:exon14:c.A920C:p.Q307P | no |
|  |  |  |  | NM_145160:exon14:c.A920C:p.Q307P | undetermined |
| 54 | ACAN | chr15 | 89386652 | NM_001135:exon6:c.G824A:p.R275Q | no |
|  |  |  |  | NM_013227:exon6:c.G824A:p.R275Q | no |
|  |  |  | 89398605 | NM_001135:exon12:c.G2789T:p.S930I | no |
|  |  |  |  | NM_013227:exon12:c.G2789T:p.S930I | no |
|  |  |  | 89398631 | NM_001135:exon12:c.T2815A:p.S939T | no |
|  |  |  |  | NM_013227:exon12:c.T2815A:p.S939T | no |
|  |  |  | 89399986 | NM_001135:exon12:c.C4170G:p.D1390E | no |
|  |  |  |  | NM_013227:exon12:c.C4170G:p.D1390E | no |
|  |  |  | 89400023 | NM_001135:exon12:c.A4207G:p.T1403 | no |
|  |  |  |  | NM_013227:exon12:c.A4207G:p.T1403A | no |
|  |  |  | 89400339 | NM_001135:exon12:c.A4523C:p.E1508A | no |
|  |  |  |  | NM_013227:exon12:c.A4523C:p.E1508A | no |
|  |  |  | 89401109 | NM_001135:exon12:c.A5293G:p.I1765V | no |
|  |  |  |  | NM_013227:exon12:c.A5293G:p.I1765V | no |
|  |  |  | 89402051 | NM_001135:exon12:c.A6235G:p.I2079V | no |
|  |  |  |  | NM_013227:exon12:c.A6235G:p.I2079V | no |
|  |  |  | 89417238 | NM_013227:exon17:c.A7499G:p.Q2500R | no |
| 55 | MYH11 | chr16 | 15820863 | NM_002474:exon28:c.G3700A:p.A1234T | no |
|  |  |  |  | NM_022844:exon28:c.G3700A:p.A1234T | undetermined |
|  |  |  |  | NM_001040113:exon29:c.G3721A:p.A1241T | undetermined |
|  |  |  |  | NM_001040114:exon29:c.G3721A:p.A1241T | undetermined |
| 56 | PKD1L2 | chr16 | 81208515 | NM_001076780:exon16:c.C2588T:p.A863V | undetermined |
|  |  |  | 81213378 | NM_001076780:exon13:c.T2132C:p.L711P | undetermined |
|  |  |  | 81232275 | PKD1L2:NM_001076780:exon7:c.C1535T:p.P512L | undetermined |
|  |  |  | 81232564 | NM_001076780:exon7:c.A1246C:p.K416Q | undetermined |
|  |  |  | 81248716 | NM_001076780:exon3:c.G547A:p.V183I | undetermined |
|  |  |  | 81249927 | NM_001076780:exon2:c.G386A:p.G129D | undetermined |
|  |  |  | 81249954 | NM_001076780:exon2:c.A359T:p.Q120L | undetermined |
|  |  |  | 81253759 | NM_001076780:exon1:c.T217C:p.W73R | undetermined |
|  |  |  | 81253917 | NM_001076780:exon1:c.T59C:p.V20A | undetermined |
| 57 | PPL | chr16 | 4933939 | NM_002705:exon22:c.C4717G:p.Q1573E | no |
|  |  |  | 4938160 | NM_002705:exon20:c.A2457C:p.R819S | no |
|  |  |  | 4945687 | NM_002705:exon10:c.C1003A:p.L335M | no |
| 58 | MYH4 | chr17 | 10346781 | NM_017533:exon40:c.A5731G:p.K1911E | undetermined |
|  |  |  | 10348354 | NM_017533:exon37:c.A5405G:p.D1802G | undetermined |
|  |  |  | 10355763 | NM_017533:exon26:c.A3318G:p.I1106M | undetermined |
| 59 | PITPNM3 | chr17 | 6406883 | NM_001165966:exon3:c.G130A:p.A44T | undetermined |
|  |  |  |  | NM_031220:exon4:c.G238A:p.A80T | no |
| 60 | KIF2B | chr17 | 51900729 | NM_032559:exon1:c.C335T:p.A112V | no |
| 61 | TTLL6 | chr17 | 46847364 | NM_173623:exon7:c.G1215T:p.E405D | no |
|  |  |  |  | NM_001130918:exon14:c.G2136T:p.E712D | no |
|  |  |  | 46894377 | NM_001130918:exon1:c.T58G:p.W20G | no |
| 62 | KRT15 | chr17 | 39670912 | NM_002275:exon7:c.C1262G:p.A421G | no |
|  |  |  | 39674641 | NM_002275:exon1:c.A439G:p.T147A | no |
| 63 | KIAA0753 | chr17 | 6515387 | NM_014804:exon8:c.T1397C:p.L466P | undetermined |
| 64 | KRTAP9-4 | chr17 | 39406291 | NM_033191:exon1:c.T319C:p.Y107H | undetermined |
|  |  |  | 39406343 | NM_033191:exon1:c.A371G:p.N124S | undetermined |
|  |  |  | 39406409 | NM_033191:exon1:c.C437A:p.S146Y | undetermined |
|  |  |  | 39406427 | NM_033191:exon1:c.T455C:p.F152S | undetermined |
| 65 | KRTAP4-1 | chr17 | 39274069 | NM_033059:exon1:c.C499G:p.R167G | no |
|  |  |  | 39274518 | NM_033059:exon1:c.G50A:p.R17Q | no |
| 66 | KIF18B | chr17 | 43004436 | NM_001264573:exon14:c.C2323T:p.P775S | undetermined |
|  |  |  |  | NM_001265577:exon14:c.C2287T:p.P763S | undetermined |
|  |  |  | 43008970 | NM_001264573:exon11:c.A1553G:p.Q518R | undetermined |
|  |  |  |  | NM_001265577:exon11:c.A1517G:p.Q506R | undetermined |
|  |  |  | 43013535 | NM_001264573:exon2:c.G178T:p.G60C | undetermined |
|  |  |  |  | NM_001265577:exon2:c.G178T:p.G60C | undetermined |
| 67 | JUP | chr17 | 39913771 | NM_002230:exon12:c.G1942A:p.V648I | undetermined |
|  |  |  |  | NM_021991:exon12:c.G1942A:p.V648I | undetermined |
|  |  |  | 39913900 | NM_002230:exon11:c.G1910A:p.R637H | no |
|  |  |  |  | NM_021991:exon11:c.G1910A:p.R637H | no |
| 68 | KRT27 | chr17 | 38933388 | NM_181537:exon8:c.T1243G:p.S415A | no |
|  |  |  | 38935812 | NM_181537:exon5:c.T914C:p.I305T | no |
|  |  |  | 38938316 | NM_181537:exon1:c.G430A:p.E144K | no |
|  |  |  | 38938591 | NM_181537:exon1:c.G155C:p.S52T | no |
| 69 | SKA1 | chr18 | 47908556 | NM_001039535:exon4:c.G271A:p.V91I | undetermined |
|  |  |  |  | NM_145060:exon4:c.G271A:p.V91I | no |
| 70 | EMR3 | chr19 | 14752325 | NM_032571:exon10:c.G1154A:p.R385Q | no |
|  |  |  | 14769339 | NM_032571:exon5:c.G379C:p.E127Q | no |
| 71 | CCDC105 | chr19 | 15133762 | NM_173482:exon7:c.G1331C:p.S444T | undetermined |
| 72 | HOMER3 | chr19 | 19040312 | NM_001145724:exon8:c.C918G:p.S306R | no |
|  |  |  |  | NM_001145721:exon10:c.C1017G:p.S339R | no |
|  |  |  |  | NM_001145722:exon10:c.C1026G:p.S342R | undetermined |
|  |  |  |  | NM_004838:exon10:c.C1026G:p.S342R | no |
| 73 | CD33 | chr19 | 51728477 | NM_001177608:exon2:c.C41T:p.A14V | undetermined |
|  |  |  |  | NM_001772:exon2:c.C41T:p.A14V | no |
|  |  |  | 51738917 | NM_001082618:exon5:c.G529A:p.G177R | undetermined |
|  |  |  |  | NM_001177608:exon6:c.G910A:p.G304R | undetermined |
|  |  |  |  | NM_001772:exon6:c.G910A:p.G304R | no |
| 74 | UMODL1 | chr21 | 43531008 | NM_001004416:exon11:c.T1676C:p.M559T | no |
|  |  |  |  | NM_001199527:exon11:c.T1460C:p.M487T | undetermined |
|  |  |  |  | NM_001199528:exon11:c.T1460C:p.M487T | no |
|  |  |  |  | NM_173568:exon11:c.T1676C:p.M559T | no |
|  |  |  | 43531403 | NM_001199527:exon11:c.C1855G:p.R619G | undetermined |
|  |  |  |  | NM_173568:exon11:c.C2071G:p.R691G | no |
|  |  |  | 43531553 | NM_001199527:exon11:c.A2005C:p.T669P | undetermined |
|  |  |  |  | NM_173568:exon11:c.A2221C:p.T741P | no |
|  |  |  | 43531632 | NM_001199527:exon11:c.T2084C:p.I695T | undetermined |
|  |  |  |  | NM_173568:exon11:c.T2300C:p.I767T | no |
|  |  |  |  | NM_001004416:exon12:c.T1916C:p.I639T | undetermined |
|  |  |  |  | NM_001199528:exon12:c.T1700C:p.I567T | undetermined |
|  |  |  | 43539287 | NM_001199527:exon14:c.G2710A:p.V904I | undetermined |
|  |  |  |  | NM_173568:exon14:c.G2926A:p.V976I | no |
|  |  |  |  | NM_001004416:exon15:c.G2542A:p.V848I | undetermined |
|  |  |  |  | NM_001199528:exon15:c.G2326A:p.V776I | undetermined |
|  |  |  | 43539293 | NM_001199527:exon14:c.G2716A:p.V906I | undetermined |
|  |  |  |  | NM_173568:exon14:c.G2932A:p.V978I | no |
|  |  |  |  | NM_001004416:exon15:c.G2548A:p.V850I | undetermined |
|  |  |  |  | NM_001199528:exon15:c.G2332A:p.V778I | undetermined |
|  |  |  | 43547873 | NM_001199527:exon19:c.G3790A:p.D1264N | undetermined |
|  |  |  |  | NM_173568:exon19:c.G4006A:p.D1336N | no |
|  |  |  |  | NM_001004416:exon20:c.G3622A:p.D1208N | undetermined |
|  |  |  |  | NM_001199528:exon20:c.G3406A:p.D1136N |  |
| 75 | KRTAP10-2 | chr21 | 45971109 | NM_198691:exon1:c.C839T:p.P280L | undetermined |
|  |  |  | 45971152 | NM_198693:exon1:c.C233T:p.S78L | no |
| 76 | SCUBE1 | chr22 | 43610207 | NM_173050:exon16:c.T1942C:p.S648P | no |
|  |  |  | 43623395 | NM_173050:exon10:c.G1192C:p.G398R | no |
| 77 | SFI1 | chr22 | 31971258 | NM_001258326:exon8:c.T718C:p.Y240H | undetermined |
|  |  |  |  | NM_001258327:exon8:c.T718C:p.Y240H | undetermined |
|  |  |  |  | NM_001258325:exon9:c.T892C:p.Y298H | no |
|  |  |  |  | NM_001007467:exon10:c.T964C:p.Y322H | undetermined |
|  |  |  |  | NM_014775:exon10:c.T964C:p.Y322H | no |
|  |  |  | 32011225 | NM_001258325:exon27:c.T3095C:p.L1032P | no |
|  |  |  |  | NM_001258326:exon27:c.T3014C:p.L1005P | undetermined |
|  |  |  |  | NM_001258327:exon27:c.T2978C:p.L993P | undetermined |
|  |  |  |  | NM_014775:exon28:c.T3167C:p.L1056P | no |
|  |  |  |  | NM_001007467:exon29:c.T3260C:p.L1087P | undetermined |
| 78 | MAP7D3 | chrX | 135310785 | NM_001173517:exon8:c.A1400C:p.E467A | undetermined |
|  |  |  |  | NM_001173516:exon9:c.A1451C:p.E484A | no |
|  |  |  |  | NM_024597:exon9:c.A1505C:p.E502A | no |
